# The Electronic Properties of Cadmium Naphthalene Diimide Coordination Complex

**DOI:** 10.3390/molecules28093709

**Published:** 2023-04-25

**Authors:** Wajid Hussain, Maroof Ahmad Khan, Zhongkui Li, Muhammad Javed Iqbal, Mubashar Ilyas, Hui Li

**Affiliations:** Key Laboratory of Cluster Sciences of Ministry of Education, School of Chemistry and Chemical Engineering, Beijing Institute of Technology, Beijing 102488, China

**Keywords:** Cd(II) coordination complex, l-alanine terminal-substituted naphthalene diimide, 28 NDI-based functional ligand, single crystal structural analysis, DFT theoretical study, electronic 29 properties

## Abstract

The computational simulations for electronic properties of cadmium (Cd) coordinated L-alanine NDI ligand (H_2_-l-ala NDI) based complex are the focus of this research. For the first time, the Cd-NDI complex (monomer) has been produced using water as the solvent; this is a new approach to synthesizing the Cd-NDI complex that has not been reported yet. Along with crystallography and Hirsch field analysis, CAM-B3LYP/LANL2DZ and B3LYP/LANL2MB basis sets were used, and in-depth characterisation of the Cd-NDI complex by following DFT and TD-DFT hypothetical simulations. Hyperpolarizabilities, frontier molecular orbitals (FMOs), the density of states (DOS), dipole moment (µ), electron density distribution map (EDDM), transition density matrix (TDM), molecular electrostatic potential (MEP), electron-hole analysis (EHA), and electrical conductivity (σ) have all been studied regarding the Cd-NDI complex. The vibrational frequencies and types of interaction are studied using infrared (IR) and non-covalent interaction (NCI) analysis with iso-surface. In comparison to the Cd-NDI complex with 2.61, 2.42 eV E_g_ (using CAM-B3LYP/LANL2DZ and B3LYP/LANL2MB basis sets, respectively) and 376 nm λ_max_, (in case of B3LYP/LANL2MB λ_max_ is higher), H_2_-l-ala NDI have 3.387 eV E_g_ and 375 nm λ_max_, metal-ligand coordination in complex dramatically altered charge transfer properties, such as narrowing band gap (E_g_). Based on the electronic properties analysis of Cd-NDI complex, it is predicted that the Cd-NDI complex will have a spectacular (nonlinear optical) NLO response. The Cd-NDI complex is discovered to be advantageous for the creation of future nanoscale devices due to the harmony between the Cd metal and H_2_-l-ala NDI, in addition to their influences on NLO characteristics.

## 1. Introduction

In 1961, Franklin and his colleagues shifted their research focus to the electrical properties field after notable advancements in the ruby laser and second harmonic generation (SHG). This led to the emergence of an interdisciplinary field that is both unique and highly competitive [1,2]. The potential applications of electrical materials, as in photonics, light emitters, optical switching, electro-opto modulations, and holographic imaging, have made them increasingly popular [3]. In the 1930s, electro-opto processes were generally ignored for thirty years while they were being proposed. Presently, optical switching, luminous materials gyroscopes [4], biosensor scanners [5], frequency mixing and SHG, are examples of recent high-tech deployments [6]. Importantly, the calculation of a large amount of data originating from many sources necessitates the use of powerful and capable computers. Electro-opto technology offers a variety of methods for increasing data handling capabilities [7,8]. To meet the growing computing needs, researchers have developed a range of current organic and inorganic electro-opto active materials [9,10].

Transition-metal complexes based on these materials have taken a prominent position due to their improved electrical and structural properties, as well as polarizability coefficient [11,12]. Due to desirable properties such as ease of synthesis, transparency, strength, and increased thermal stability, π-conjugated metal-organic compounds along amidic group-based electro-opto materials with significant first or second hyperpolarizabilities had piqued researchers’ interest [13,14]. To obtain ultrarapid high hyperpolarizability response, a wide range of conventional techniques have been used, including donor–acceptor bridge system [15,16], diradical character [17], octupolar molecules [18], and excess electron model.

By optimizing the molecular design, it is possible to increase the potential applications of π-conjugated materials in exciting fields such as microelectronics, structural properties, electrochemistry, and optoelectronics [19,20,21,22,23]. However, to the best of our knowledge, the effect of metal coordination has yet to be accounted for. As a result, the purpose of this study is to examine the effects of Cd coordination with H_2_-l-ala NDI on optoelectronic properties. For a long time, we have been focusing on functional-supramolecular coordination chemistry for a wide range of applications. Recent research has looked into coordination complexes between NDI (naphthalene diimides) and PDI (perylene diimides) [24,25]. As part of a study inspired by the systematic exploration of the electronic properties of Cd-NDI complex, a crystallographic study was conducted. Moreover, Cd-NDI complex and H_2_-l-ala NDI were simulated using the CAM-B3LYP/LANL2DZ and B3LYP/LANL2MB basis sets, aiming to achieve high NLO response.

## 2. Results and Discussion

### 2.1. Crystal Structure of Complex

H_2_-l-ala NDI can create catenane motifs, even though the ligands can also acquire an unusual conformation that precludes the formation of cyclic motifs. l-phenyl alanine (bulky amino acids) is not capable of catenane production, although they do have a proclivity for rotaxanes [26,27]. For complexation, Li et al. used γ-aminobutyric acid naphthalene diimide derivative (H_2_GABA-NDI). This ligand is lengthier and consequently more flexible than H_2_-l-ala NDI. The H_2_GABA-flexibility NDIs makes it easier for it to release its terminal or carboxylic acid protons, making coordinated bonding with any metal easier. However, because the amino group is located at longer distance, the ligands are stiffer and far less flexible. Albeit, when their length decreases, their metal coordinated complexes become tighter in the packing scenario.

**Figure 1:**
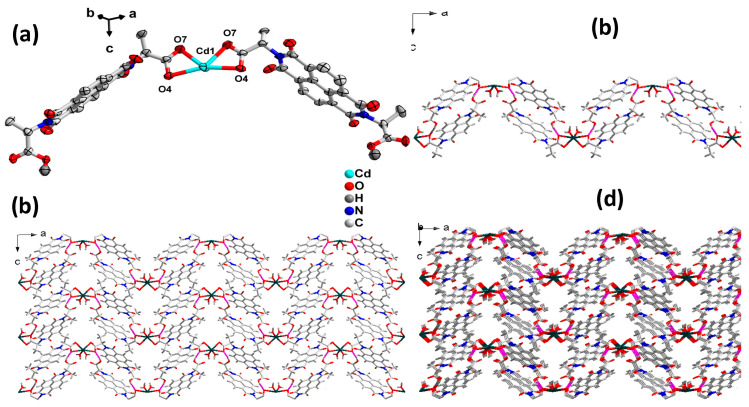
The ORTEP picture of Cd-NDI complex showing the coordination environments of Cd (II) (**a**). One-dimensional crystal structure of Cd-NDI complex (**b**). Two-dimensional crystal structure of Cd-NDI complex (**c**). Three-dimensional crystal structure of Cd-NDI complex (**d**).

Interestingly, the H_2_-l-ala NDI may create a self-interdependent catenane motif when coupled to Cd metal (monomer). The single-crystal X-ray diffraction method was used to study the yellow-colored crystal of the Cd-NDI complex (CCDC number 2171360). *P2*_1_*2_1_2* space group with distorted octahedral coordination geometry was discovered in the coordination environment of the Cd-NDI complex (Figure 1a and Table 1).

The carboxylate and hydroxyl group of H_2_-l-ala NDI endorse the wings-like conformation by acting Cd^2+^ ions as a bridge (through C=O and O-H oxygen atoms) between them, which gives rise to 1D chain (Figure 1b). Here, Cd^2+^ is coordinated by six oxygen atoms that belong to carboxylate and hydroxyl group of H_2_-l-ala NDI. The NDI carboxylic acid oxygens (O1, O3, O8, and O9). The O-H group of both H_2_-l-ala NDI (of different layers) are bonded through hydrogen bond (2.584 Å) that elongate on *x*-axis are responsible for 2D coordination geometry (Figure 1c, Table 2). Two-dimensional geometry makes a complex more stable than 1D (stated, higher electrical/thermal stability, larger specific surface area, and lower sheet agglomeration are the stated advantages of a 2D chain over 1D 41). The bond lengths of Cd–O7, Cd–O4, and Cd–O2, are 2.276 Å, 2.512 Å, and 2.171 Å, respectively. Nevertheless, van der Waals forces and weak π-π stacking interaction (6.22 Å) are responsible for the 3D structure of the complex.

**Figure 2:**
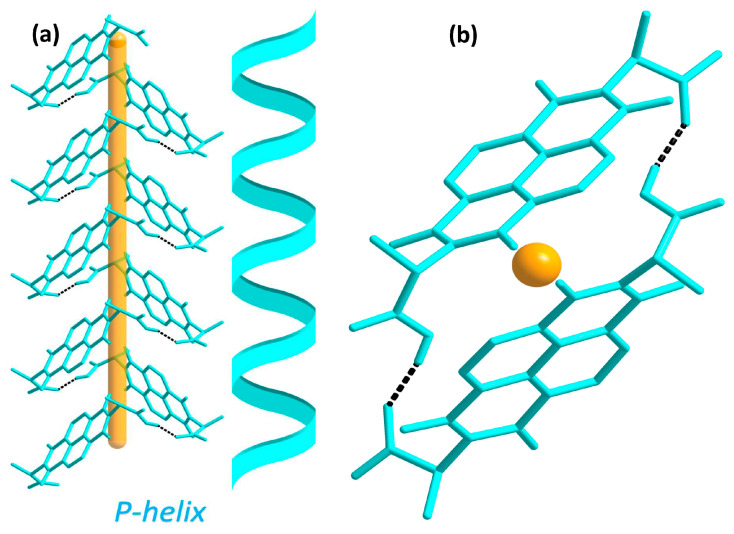
Helical structure of Cd-NDI complex through HB (**a**). Top view of Cd-NDI P-helix (**b**).

Moreover, H_2_-l-ala NDI has inherent chirality that also generates chirality in the Cd-NDI complex through hydrogen bonds (Figure 2a). This chirality enables the complex to form a helical structure, as shown in Figure 2b.

The thermogravimetric graphs of the Cd-NDI complex that have revealed thermal stability are shown in Figure S3. Under Ar gas, the Cd-NDI complex was subjected to thermogravimetric analyses (TGA) and differential scanning calorimetry (DSC). In the instance of the Cd-NDI complex, the elimination of H_2_O, CO_2_, NO_2_, and coordinated solvent caused a weight loss of around 40% from ambient temperature to 400 °C. The decrease of weight at 530 °C was due to the loss of organic linkers and weakening of the framework, while the Skelton was fairly resilient between 400 and 530 °C. A stiff π-conjugated system of NDI with a carboxylate group, bond energy, interpenetrated arrangement, or a strong π-stacking network, could all contribute to thermal stability.

### 2.2. Hirschfield Surface Analysis

**Figure 3:**
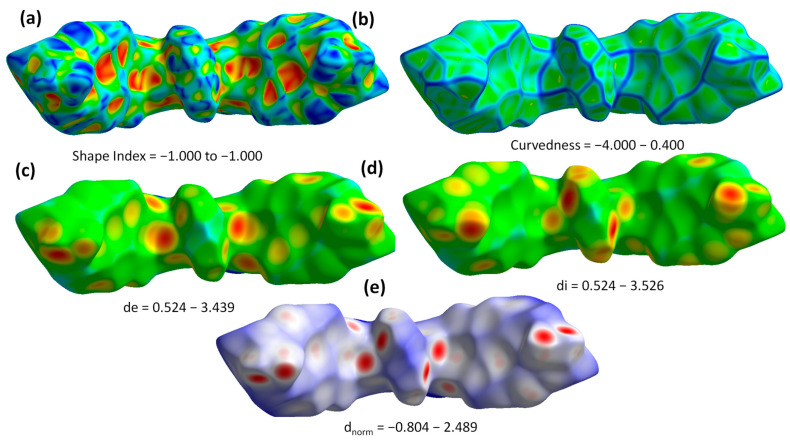
Hirschfield surface maps of Cd-NDI complex over: (**a**) shape index; (**b**) curvedness; (**c**) de; (**d**) di; and (**e**) d_norm_, respectively.

Hirschfield surface analysis was performed to study the weak interactions in the complex, such as hydrogen bond, π-π interaction, molecular packing in crystal, and van der Waals interactions. In this study, the donor/acceptor moieties are denoted by the blue convex, and red concave regions, respectively, that are studied with shape index in Figure 3a. The Hirschfield analysis stands out for having a new crystal-structure, which increases the possibility of learning more about the crystal. Using HSs made it possible to evaluate several crystal properties quantitatively, including area (SH), volume (VH), sphericity (Ω), and globularity (G). The certainty of a uniform surface with form index values in 1.000 to 1.000 range is revealed by the value of curvedness, which ranges from 4.000 to 0.400. This number shows a positive divergence from the standard mean position (Figure 3b). The d_norm_ and cool contact detachment provide a comprehensive relationship between the distances of any superficial point to the surrounding internal (di) and exterior (de) atoms along the van der Waals distance. The complex HSs are shown in Figure 3c,d along with their d_norm_, di, and de values. The d_norm_ map in Figure 3e is depicted with distinct hues ranging from 2.489 (blue) to 0.804 (red). As seen by the red area in Figure 3d, the hydrogen bonding between O-H is the most important interaction and is what causes the lower values of di and de in comparison to the van der Waals radii. While the white area shows an identical distance, the blue zone shows a higher distance than the van der Waals radii. The computation of the high and low electron density zones was made possible through map analysis. The colors blue and red represent the electrostatic potentials surrounding the atoms, with blue denoting favorable potentials and red denoting unfavorable potentials. The oxygen atom’s electronegative zone and the C-H bonds’ electropositive region were both visible within the molecule.

### 2.3. Optimized Geometries

**Figure 4:**
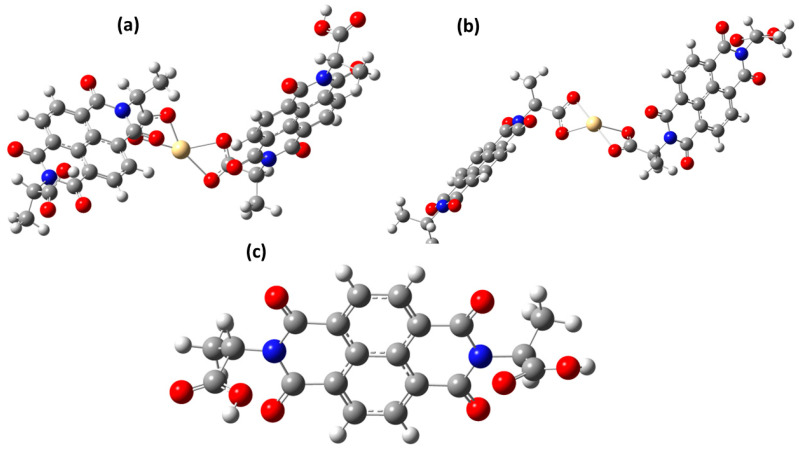
Optimized geometries of: (**a**) Cd-NDI complex through CAM-B3LYP/LANL2DZ; (**b**) Cd-NDI complex through B3LYP/LANL2MB; and (**c**) H_2_-l-ala NDI through CAM-B3LYP/LANL2DZ, respectively.

Using CAM-B3LYP/LANL2DZ (Figure 4a) and B3LYP/LANL2MB (Figure 4b) basis sets, Cd-NDI complex and NDI ligand (Figure 4c) geometries were optimized (Figure 4). By acting as a bridge between NDI core, the carboxylate and hydroxyl group of H_2_-l-ala NDI support the wings-like conformation of the complex in both optimized geometries (Figure 4a,b). Six oxygen atoms from the carboxylate and hydroxyl groups of H_2_-l-ala NDI are used to coordinate Cd^2+^ in this instance (two were eliminated for clarity during crystal structure formation).

### 2.4. Photophysical Properties

Figure S4a illustrates the UV absorption spectra of Cd-NDI complex and H_2_-l-ala NDI in DMF. In the case of the Cd-NDI complex, the intensity of the absorption peaks with maxima at 325 (minor peak was seen), 330, 355, and 375 nm was observed (Figure S4a). In the case of H_2_-l-ala NDI, three peaks with small intensities were identified (with only one peak showing a slight change, Figure S4a). In any case, Cd-NDI complex shows two distinct absorption bands: the first, at 300–350 nm, is a high-energy band that is exempted owing to the π−π* transition; the second, about 300–400 nm, is a low-energy band associated to intramolecular charge transfer (ICT).

When studying electronic excitations and charge transfer, itis vital to look at UV-visible spectra. In the pure phase, the absorption spectra of Cd-NDI complex and H_2_-l-ala NDI is also estimated using the CAM-B3LYP/LANL2DZ and B3LYP/LANL2MB basis sets. As shown in Figure S4b–d, the absorption spectra of Cd-NDI complex and H_2_-l-ala NDI show that in the pure phase, Cd-NDI complex has a higher wavelength than H_2_-l-ala NDI (d). Using CAM-B3LYP/LANL2DZ basis set, λ_max_ of the complex is lower than using B3LYP/LANL2MB set. Regardless, the use of pure phase shifting toward higher wavelengths was discovered to be attributable to the stabilisation of π-electrons [28]. In any case, the shift in peak position (from experimental to computational) could be related to the transition from the liquid to the pure phase of the medium.

As we know there is a strong relationship between λ_max_ and band gap (E_g_), electronic excitation is possible with a lower E_g_; since it is energetically advantageous to remove electrons from low-energy HOMO, or add electrons to high-energy LUMO, resulting in the production of activated complexes (as narrow E_g_ is frequently connected to low kinetic stability). These coordinated complexes can be classified as n-type semiconductors due to their lower E_g_ and have been termed “new members of the NLO family” [29]. In both experimental and pure phase studies, the λ_max_ value of the Cd-NDI complex is 375 and 475, respectively (Figure S4). However, the use of pure phase redshifted the λ_max_ of the Cd-NDI complex. Due to the stabilisation of delocalized π-electrons, the λ_max_ of Cd-NDI complex and H_2_-l-ala NDI (experimentally) is 375 and 374 nm, respectively. Regardless, the photophysical features exhibited by the Cd-NDI complex have been linked to a λ_max_ due to the complex’s extended conjugation.

### 2.5. Frontier Molecular Orbitals (FMOs)

**Figure 5:**
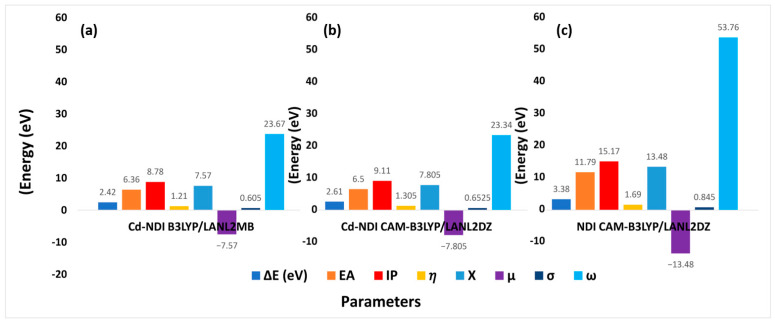
E_g_, IP, EA, hardness (η), electronegativity (X), chemical potential (µ), global softness (σ), and electrophilicity (ω) of Cd-NDI complex (**a**) through B3LYP/LANL2MB, (**b**) through CAM-B3LYP/LANL2DZ and (**c**) H_2_-l-ala NDI through CAM-B3LYP/LANL2DZ, respectively.

**Figure 6:**
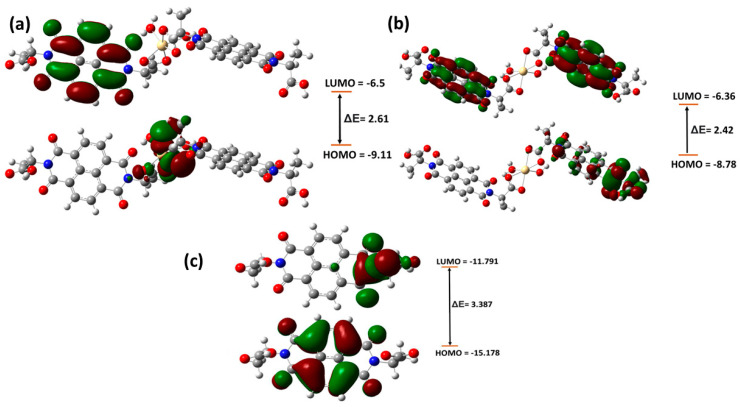
(**a**) FMO plots of Cd-NDI complex through CAM-CAM-B3LYP/LANL2DZ; (**b**) through B3LYP/LANL2MB; (**c**) H_2_-l-ala NDI through CAM-B3LYP/LANL2DZ and band gaps (E_g_), respectively.

The analysis of molecule FMOs is a crucial step in determining charge mobility [30]. Itis also useful for explaining charge transfer and distribution patterns in coordination systems. The absorption features and electronic characteristics of molecular systems significantly depend on the lowest unoccupied (LUMO) and highest occupied (HOMO) energies [31]. LUMO only has high energy charges, whereas HOMO has a condensed population electronic charge, analogous to the conduction and valence bands in band theory [32]. For both the Cd-NDI complex and the H_2_-l-ala NDI, the HOMO and LUMO energies, as well as their E_g_, are studied, and their values are illustrated in Figure 6. Using CAM-B3LYP/LANL2DZ and B3LYP/LANL2MB, the HOMO of the Cd-NDI complex is −9.11 and −8.78 eV and the LUMO is −6.5 eV, −6.36 eV, respectively. While the HOMO of the H_2_-l-ala NDI is −15.178 and the LUMO is −11.791 eV, respectively. The E_g_ governs charge mobility in practice. Lower E_g_ permits the charge to be excited and transmitted across the π-conjugated system more efficiently. Electrical conductivity and E_g_ are inextricably related. When compared to H_2_-l-ala NDI (3.387), E_g_ of Cd-NDI complex systems is narrow and lies at 2.62 eV (CAM-B3LYP/LANL2DZ) and 2.42 eV (B3LYP/LANL2MB). Increased conduction capability is confirmed by a narrow E_g_ in the Cd-NDI complex. Cd-NDI complex is considered polarizable and efficient charge transfer complex is due to its low E_g_ value. Figure 5 and Figure 6 depict the values of HOMOs, LUMOs, and E_g_ values.

Stability is an important aspect in any complex, along with electrical conduction. As a result, the stability of the Cd-NDI complex was calculated. Chemical potential (μ) amplitudes, which have been computed, are frequently used to explain complex stability. As we all know, the moiety with the highest chemical potential (μ) is more stable than the moiety with the lowest chemical potential. The chemical potential (μ) values of the Cd-NDI complex are −7.57 μ (CAM-B3LYP/LANL2DZ) and −7.80 μ (B3LYP/LANL2MB). While ligands have −13.4849 μ, respectively, that concluded complex is more stable than ligand (Figure 5).

### 2.6. Density of States (DOS)

**Figure 7:**
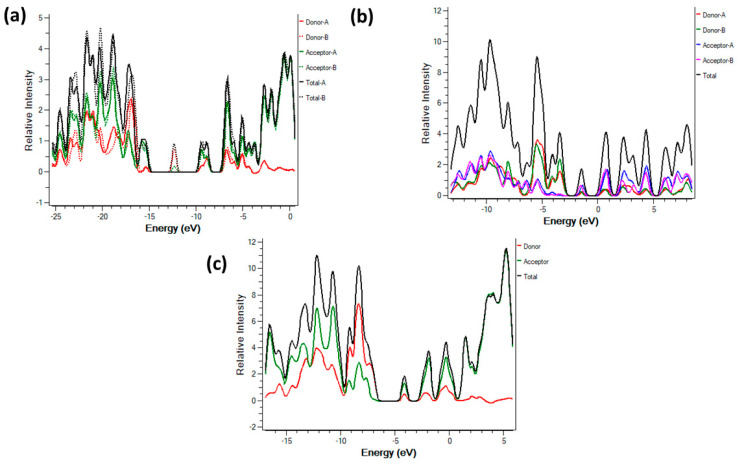
(**a**) DOS charts of Cd-NDI complex through CAM-B3LYP/LANL2DZ; (**b**) through B3LYP/LANL2MB; (**c**) H_2_-l-ala NDI through CAM-B3LYP/LANL2DZ, respectively.

DOS is a method to analyze charge transport occupancy of states using an analytical technique. Additionally, the Mulliken charge distribution has backed up the FMO findings and how the fragmentation of molecules enhances the HOMO and LUMO molecular orbitals, according to DOS study [33]. DOS approximations can also be used to calculate HOMO and LUMO energies. DOS plots have been shown to help comprehend the role of complexes and their fragments in chemical band gap research. Figure 7a,c depicts the DOS graphs of both complex and ligand that were obtained using CAM-B3LYP/LANL2DZ and B3LYP/LANL2MB basis sets. Moreover, the total contributions of donors and acceptors are given in Table S10. Using CAM-B3LYP/LANL2DZ in the formation of LUMO in the Cd-NDI complex (Figure 6a) and H_2_-l-ala NDI (Figure 6c), HOMO is acquired primarily by the acceptor atoms and insignificantly by the donor atoms, whereas in the formation of LUMO in the H_2_-l-ala NDI, donor plays a vital role, whereas acceptor plays a vital role in the Cd-NDI complex. Additionally, the d orbital is the main contributor to the conduction band in the range of 0.0 eV to 10.0 eV due to s, p, d, orbitals overlap of N, O, and Cd atoms. Moreover, while using B3LYP/LANL2MB set, almost the same trend has been observed (somewhere donor atoms take the charge). Acceptor is playing a vital role in the formation of FMOs that lead to help in the electronic study (Figure 7b).

### 2.7. Dipole Moment (µ)

In crystallinity and molecular packing, the dipole moment (μ) plays a vital role. Dipole moment is calculated using the CAM-B3LYP/LANL2DZ and B3LYP/LANL2MB basis sets for the Cd-NDI complex and the H_2_-l-ala NDI. The Cd-NDI complex acquired a higher dipole moment (5.36 D (CAM-B3LYP/LANL2DZ) and 7.86 D (B3LYP/LANL2MB)) than the H_2_-l-ala NDI (1.14 D), according to the findings. Due to the symmetry in its structure, H_2_-l-ala NDI has a lower µ 1.14 D. The Cd-NDI complex, which combines a Cd atom with two H_2_-l-ala NDI wings to promote self-assembly and multilayer manufacturing, had the highest value of µ (5.36 D and 7.86 D). As a result, considerable NLO characteristics are possible. Similarly, the large change in μ implies that complex has arranged itself to prevent charge recombination.

### 2.8. Transition Density Matrix (TDM)

**Figure 8:**
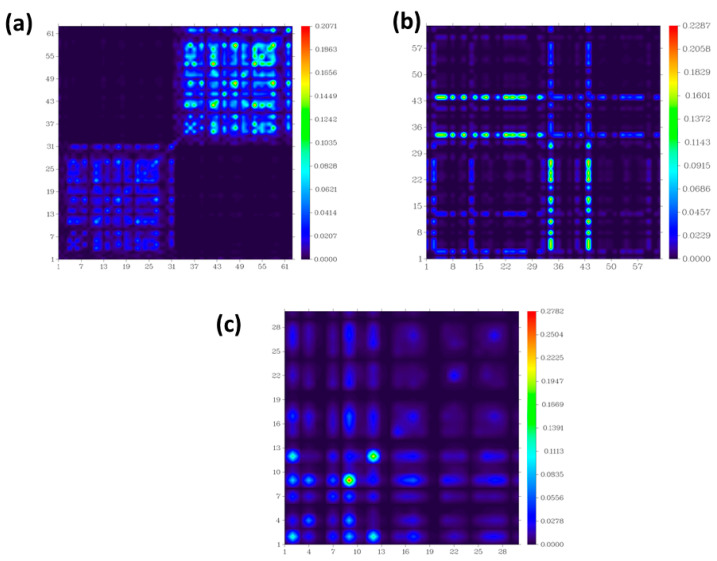
TDM plots of Cd-NDI complex: (**a**) calculated through CAM-B3LYP/LANL2DZ; (**b**) through B3LYP/LANL2MB; and (**c**) H_2_-l-ala NDI through CAM-B3LYP/LANL2DZ, respectively.

TDM is a matrix that stores information about quantum geometry and charge transition sites of the excited state of coordinated molecules [34,35]. In TDM plots, the number of atoms is depicted on the *x*-axis and *y*-axis (left side) while the coefficient of transition or electron density is displayed on the right side of the *y*-axis. TDM study of the Cd-NDI complex and H_2_-l-ala NDI has been performed using the CAM-B3LYP/LANL2DZ and B3LYP/LANL2MB functionals. Hydrogen atoms have been disregarded due to their minor contributions to transitions. Figure 8 depicts TDM for both the Cd-NDI complex (a) and the H_2_-l-ala NDI (b) using CAM-B3LYP/LANL2DZ set. While Figure 8b displays TDM of Cd-NDI complex using B3LYP/LANL2MB functional. H_2_-l-ala NDI is made up of only one component, whereas the Cd-NDI complex is divided into two parts: surface and coordination.

Charge coherency and distribution can be achieved in the Cd-NDI complex through valuable off-diagonal and diagonal charge transfer. Electronic distribution is concentrated on coordinating sections of the Cd-NDI complex. In TDM, the bright areas indicate the atom number where the transition population was found. Charge transfer improved in the Cd-NDI complex in both cases (CAM-B3LYP/LANL2DZ and B3LYP/LANL2MB functionals). As we can see, more bright areas and diagonal lines can be found in the case of CAM-B3LYP/LANL2DZ set as compared to B3LYP/LANL2MB set. When compared to H_2_-l-ala NDI, the Cd-NDI complex exhibits excellent charge separation capability, as TDM in Figure 8c has no clear diagonal line and less bright areas, as a result, the complex can be employed as unintentional candidates for future NLO materials with improved properties.

### 2.9. Molecular Electrostatic Potential Map (MEP) Analysis

**Figure 9:**
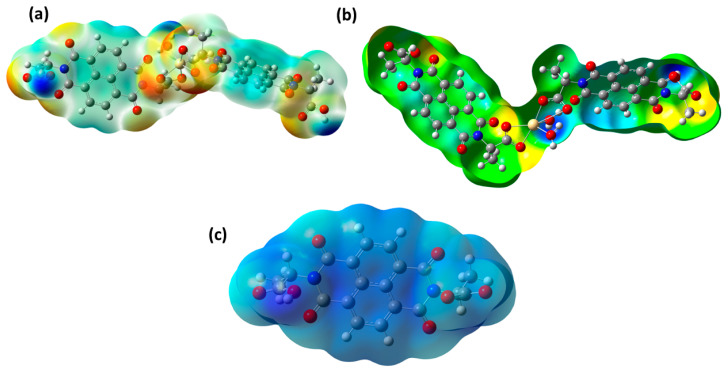
MEP images of Cd-NDI complex: calculated (**a**) through CAM-B3LYP/LANL2DZ; (**b**) through B3LYP/LANL2MB; and (**c**) H_2_-l-ala NDI through CAM-B3LYP/LANL2DZ, respectively.

**Figure 10:**
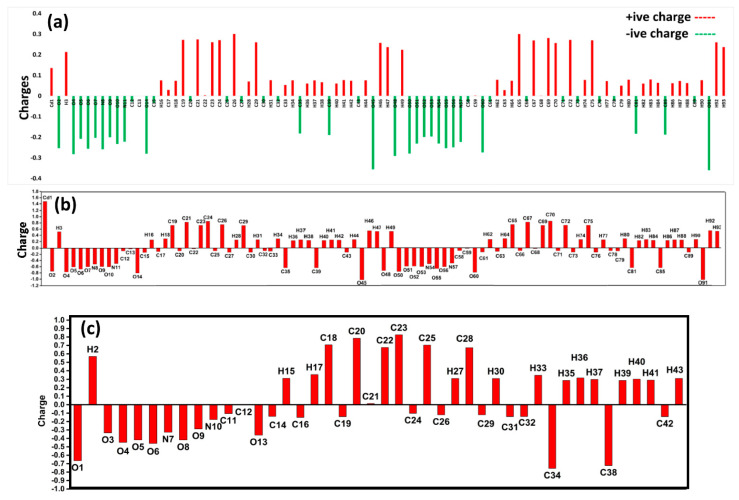
Natural population graphs of Cd−NDI complex: (**a**) through CAM-B3LYP/LANL2DZ; (**b**) through B3LYP/LANL2MB; (**c**) H_2_−l−ala NDI through CAM−B3LYP/LANL2DZ, respectively.

Based on the distribution of the Milliken charge across the whole molecule, MEP analysis captures information on atom reactivity and the presence of reactive sites for nucleophilic and electrophilic interaction [17]. MEP is also important for anticipating the relationship across molecular interaction, molecular structure, and molecule photophysical characteristics (electronic transitions) [36]. MEP simulations at optimal CAM-B3LYP/LANL2DZ (Figure 9a) and B3LYP/LANL2MB (Figure 9b) functional are used to determine the electrophilic and nucleophilic areas of the Cd-NDI complex and H_2_-l-ala NDI (Figure 9c). The nucleophilic, electron-rich, and negative MEP zone is shown in red in MEP diagrams. On comparing the red color to the natural population, the green hue signifies the neutral zone, and the blue hue suggests the electrophilic, electron-deficient, and positive MEP zone [30]. The red color (negative potential) is concentrated on the C=O and O-H regions of the Cd-NDI complex surface, making it more nucleophilic, as shown in the MEP diagram (Figure 9a,b). The imide moiety’s methyl groups (-CH_3_) and the hydrogen atoms in naphthalene exhibit a blue hue (positive potential) and act as an electrophile. From MEP we can also discuss the natural population (especially for Cd-NDI complex). The upward atoms are positively charged, while the downward mentioned are negatively charged (Figure 10a,b) CAM-B3LYP/LANL2DZ and B3LYP/LANL2MB, respectively, for Cd-NDI complex and (c) for H_2_-l-ala NDI. Moreover, MEP and natural population analysis are also helpful to study the charge transfer properties. Figure 10 reveals that the upward peaks denote the positive charged species while downward peaks indicate negative charged atoms that suggest the electrophile and nucleophile in MEP diagram (Figure 9), respectively.

### 2.10. Electron Density Distribution Matrix (EDDM)

**Figure 11:**
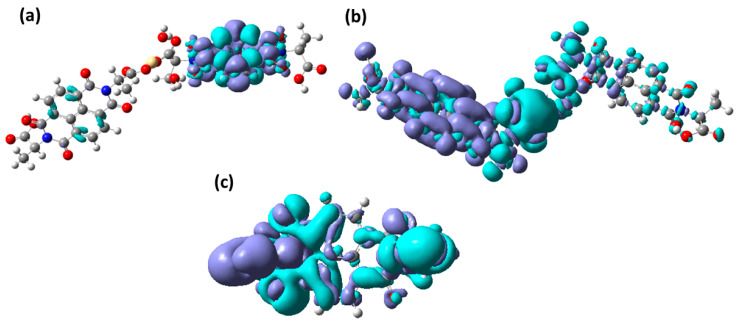
Electron density distribution matrix (EDDM) of Cd-NDI complex: (**a**) through CAM-B3LYP/LANL2DZ; (**b**) through B3LYP/LANL2MB; (**c**) H_2_-l-ala NDI through CAM-B3LYP/LANL2DZ, respectively.

To better understand the charge transfer capacity, EDDM evaluations were carried out, in which the excited state’s molecular orbitals were subtracted from the ground state. At the CAM-B3LYP/LANL2DZ and B3LYP/LANL2MB functional basis sets [37], EDDM of Cd-NDI complex and H_2_-l-ala NDI are computed. The rearrangement of electrons in the Cd-NDI complex and H_2_-l-ala NDI is depicted in EDDM plots. The net charge density of the Cd-NDI complex and H_2_-l-ala NDI is shown in Figure 11a–c. In EDDM diagrams, the sea-green hue represents a positive charge density value (also represented as upper peaks in Figure 10 in natural population analysis and blue color in Figure 9 in MEP analysis), whereas the purple color represents a negative charge density value and lower electron density following excitation (also signified as downward peaks in Figure 10 in natural population analysis and red color in Figure 9 in MEP analysis). The charge distribution pattern in the Cd-NDI complex is analogous through H_2_-l-ala NDI (since charge distribution is predominantly focused here), in that charge density is distributed uniformly across the system. If we compare EDDM study through CAM-B3LYP/LANL2DZ and B3LYP/LANL2MB, it can be seen that the charge is distributed over NDI core in the case of CAM-B3LYP/LANL2DZ (Figure 11c) while the charge is distributed almost over the whole monomer in the case of B3LYP/LANL2MB (Figure 11b).

### 2.11. Non-Covalent Interaction (NCI) Analysis

**Figure 12:**
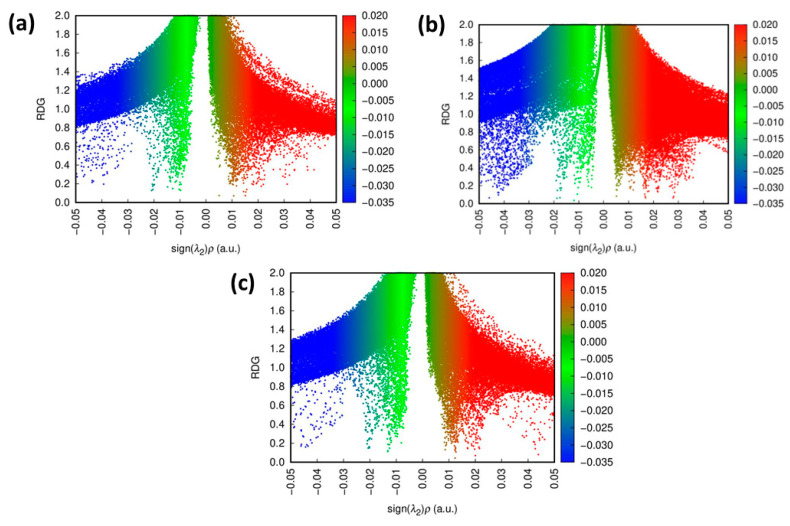
Non-covalent interaction (NCI) of Cd-NDI complex: (**a**) through CAM-B3LYP/LANL2DZ; (**b**) through B3LYP/LANL2MB; (**c**) H_2_-l-ala NDI through CAM-B3LYP/LANL2DZ, respectively.

The non-covalent interactions of a molecule can be determined using the NCI evaluation. The molecular areas with weak interactions and those with strong directional attractions associated with localised atom–atom links can be distinguished using the NCI plot [38]. The scatter graphs between the reduced electron density (r), density gradient, orientated by the sign of λ_2_, are shown in Figure 12a–c [39]. This NCI study was performed using CAM-B3LYP/LANL2DZ and B3LYP/LANL2MB basis set for Cd-NDI complex and ligand. NCI analysis appears to be a better option for improving the information on molecular stability in small or large molecules by combining data on van der Waals forces, hydrogen bonding, and steric hindrance inside a system [39]. Multiwfn software was used to create the scatter graphs.

**Figure 13:**
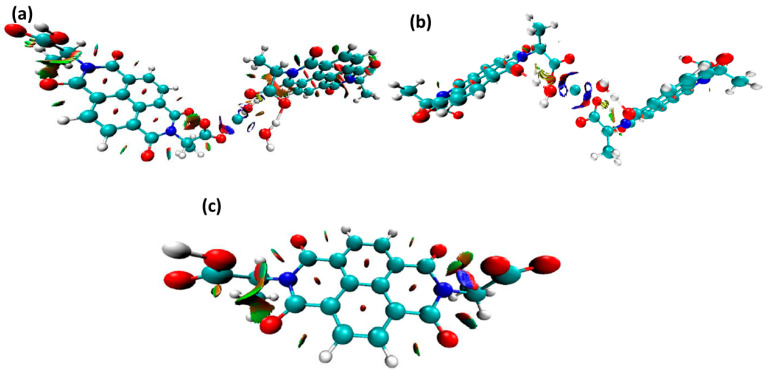
Pictorial representation of 3D iso-surface of Cd-NDI complex: (**a**) through CAM-B3LYP/LANL2DZ; (**b**) through B3LYP/LANL2MB; (**c**) H_2_-l-ala NDI through CAM-B3LYP/LANL2DZ, respectively.

The (λ_2_) values can be utilised to determine the nature of an interaction: if (λ_2_) ρ > 0, the interaction is repulsive; if (λ_2_) ρ < 0, the interaction is attractive, as shown by the color-filled 3D iso-surface of the Cd-NDI complex (Figure 13a and H_2_-l-ala NDI Figure 13c). The NCI graphs of the Cd-NDI complex (Figure 12a) and H_2_-l-ala NDI (Figure 12b) demonstrate that there are significant attractive forces between the complex molecules, which is beneficial for forming favorable NLO materials. The H-bond (attractive) is shown by the blue tone (H_2_O forms a hydrogen bond with Cd metal, leading to chirality; see crystallography section), the van der Waals interactions are represented by the green hue, and the steric effect (due to H_2_-l-ala NDI CH_3_) is represented by the red region (repulsive). If we compare CAM-B3LYP/LANL2DZ and B3LYP/LANL2MB sets, we can conclude that more blue tone is NCI graph in Figure 12b as compared to Figure 12a. Hence, more blue patches can be seen between Cd and NDI moiety (Figure 13b as compared to Figure 13a).

### 2.12. Infrared (IR) Analysis

The features of the infrared (IR) spectra of the Cd-NDI complex and H_2_-l-ala NDI (Figure S5a experimental) are investigated computationally (Figure S5b–d) to examine how they vary in coordination. Since the peak of about 3300 cm^−1^ has vanished in the Cd-NDI complex spectrum, it is responsible for Cd coordination with H_2_-l-ala NDI. Moreover, if we compare CAM-B3LYP/LANL2DZ and B3LYP/LANL2MB sets, we can see that peaks are shifted at higher frequency (cm^−1^) for B3LYP/LANL2MB in Figure S5b.

### 2.13. Cyclic Voltammetry (CV)

**Figure 14:**
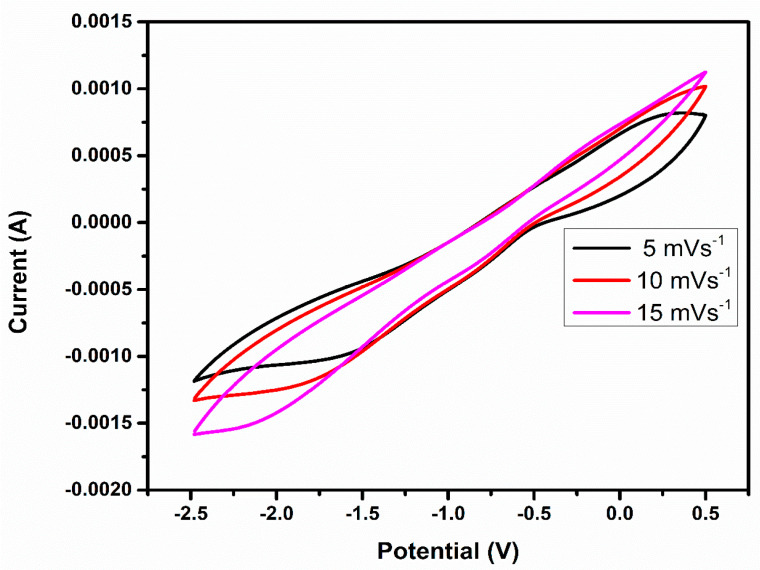
CV spectra of Cd−NDI complex at 5–15 mVs^−1^ scan rates.

To prepare the working electrode, a mixture was created by combining 80 wt% active material, which consisted of Cd-NDI complex, with 10 wt% conductivity agent (acetylene black, Super P), and 10 wt% polyvinylidene difluoride (PVDF) as a binder, using a mortar and pestle. A small amount of ethanol was then added to the mixture to create a slurry, which was pasted onto a nickel foam (1 cm × 1 cm). The electrodes were then dried at 80 °C for 12 h and pressed at 15 MPa before being created. A three-electrode cell was used to analyze the electrochemical behavior of the working electrodes, with Ag/AgCl (E_Ag_/AgCl = 0.222–0.059 log AgCl) and platinum wires being used as reference and counter electrodes. The electrodes were subjected to perform CV in 0.5 M DMF 0.38 g TBAPF_6_ electrolytes. Figure 14 shows the CV curves of Cd-NDI complex at 5–15 mVs^−1^ (redox peaks are visible at 5 mVs^−1^). Noticeable peaks ranging from −2.5–1.0 V (vs Ag/AgCl) [40] were observed in the electrochemical performance of the electrode, indicating Faradic redox reactions. The integral area of the redox peak was significantly greater than the rectangular portion of the curve, suggesting that the Faradaic pseudocapacitance primarily contributes to the capacitive property of electrode material. Moreover, Cd-NDI complex revealed clear oxidation peaks at 0.24 V, 0.3 V and 0.48 V for 5 mVs^−1^, 10 mVs^−1^ and 15 mVs^−1^_,_ respectively. While the reduction peaks appeared at −1.5 V, −1.8 V and −2.2 V for 5 mVs^−1^, 10 mVs^−1^ and 15 mVs^−1^_,_ respectively. Hence, the CV peaks of the Cd-NDI complex exhibited remarkable electrochemical reversibility that is in good agreement for electronic properties.

#### 2.13.1. Electrical Conductivity (σ)

E_g_ beside CV is the most essential aspect to consider when determining the electrical conductivity (σ) of coordinated complexes for NLO properties. Due to their narrow E_g_, Cd-NDI complex becomes more conductor- or semiconductor-like after complex formation. As a result, it is expected that the σ is greater than the ligand and can be calculated using Equation (1) [41].

σ α exp(−E_g_/2kT) (1)

Here, E_g_ signifies the band gap, k represents the Boltzmann constant, and T signifies the temperature in kelvin. Since the equation is exponentially connected to E_g_, it is predicted that the complex with lower E_g_ will have a higher value [42]. In comparison to H_2_-l-ala NDI 3.387 eV, the Cd-NDI complex has a lower value (2.62 eV and 2.42 eV for CAM-B3LYP/LANL2DZ and B3LYP/LANL2MB sets, respectively), resulting in a considerable increase in electrical conductivities of Cd-NDI complex. Strong coordination interaction and efficient charge transfer have increased σ. As a result, our produced Cd-NDI complex could act as a charge transfer material.

#### 2.13.2. Electronic Transition of NDI and PDI Ligands

Approximately all computed electronic transitions are from σ to σ* but some are from lone pair to lone pair*. These transitions are also calculated using CAM-B3LYP/LANL2DZ and B3LYP/LANL2MB sets. Cd-NDI complex has 376 nm (390 λ_max_, CAM-B3LYP/LANL2DZ and 820 λ_max_, B3LYP/LANL2MB) H_2_-l-ala NDI have 375 nm (330 CAM-B3LYP/LANL2DZ) λ_max_ (Figure S4). All the transition data are given in supporting Tables S11 and S12. It is also stated that the peaks (region) between 560–540 nm show both intramolecular charge transfer (ICT) and local excited PDI (LE) characteristics [43,44]. Another finding is the ICT from HOMO to LUMO with the π-π* character for transitions between 445 and 382 nm directed from the donor O and N to the perylene core. While the LE to MLCT charge transfer belongs to 480–485 λ_max_. The λ_max_ below 480 nm belongs to MLCT to ICT. Therefore, it can be seen that Cd-NDI complex has more transitions with less energy as compared to NDI ligand.

**Figure 15:**
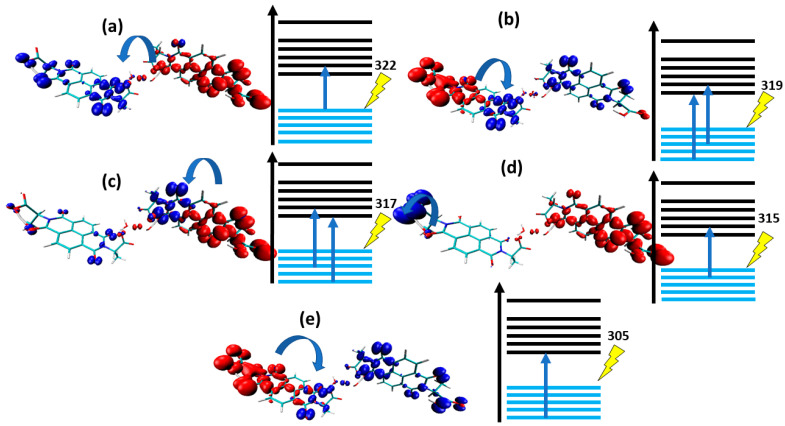
Electron-hole analysis of Cd-NDI complex (1–5 excited states, (**a**–**e**)) through CAM-B3LYP/LANL2DZ.

**Figure 16:**
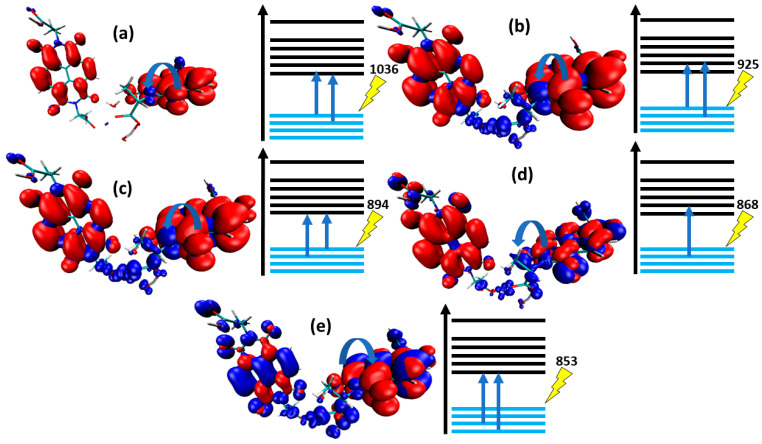
Electron-hole analysis of Cd-NDI complex (1–5 excited states (**a**–**e**)) through B3LYP/LANL2MB.

By assessing the electrical excitation of a Cd-NDI complex, one can determine its transition properties. The electron-hole hypothesis is based on the molecular-orbital theory. The contributions of the hole and electron orbitals in the singlet-excited state can be determined using this electron-hole theory, as well as the electron transfer process. According to the information in Table 3 and Table 4, Figure 15 and Figure 16 depict the electron and hole orbitals of the Cd-NDI complex for the first five excited states using two different basis set. The findings show that HOMO is present in amino acid (in both sets) and that LUMO is still present in perylene moiety (also in both sets). The λ_max_ of the Cd-NDI complex is 322.9 nm and 1036.6 nm.

Greater overlap integral (S) values between electron-hole orbitals produce faster charge transfer durations than greater overlap distances (D) between electron-hole orbitals do. According to the electron-hole orbital distance, D is greater for the first five excited states 12.651 Å, 7.416 Å, 5.090 Å, 20.768 Å, and 10.744 Å, respectively, which denotes a lengthy charge transfer length (B3LYP/LANL2MB). However, D is 4.251, 1.111, 1.119, 6.158, and 1.686 by using CAM-B3LYP/LANL2DZ set. It can be seen while using CAM-B3LYP/LANL2DZ set D values are lower than B3LYP/LANL2MB set.

For the first five excited states, the orbitals overlap integral (S) is lower (0.17161, 0.39821, 0.41036, 0.01619, and 0.31276; and 0.19131, 0.53443, 0.53190, 0.25832, and 0.39650), indicating a quick charge transfer rate in both sets because the hole and the electron orbitals are distinct. Moreover, several excited states are given in Figure 15 and Figure 16 (for B3LYP/LANL2MB and B3LYP/LANL2DZ basis set, respectively) that illustrate that at λ_max_ 1036 (1st state) two transitions occurred, one from ground state HOMO-0 to LUMO+0 and 2nd from HOMO-1 to LUMO+0. While on λ_max_ 853 (5th state), two transitions also occurred, HOMO-3 to LUMO+1 and HOMO-4 to LUMO+1. While for the 2nd state at λ_max_ 925 HOMO-0 to LUMO+1 and HOMO-1 to LUMO+1 transitions occurred. For 3rd state at λ_max_ 894 HOMO-1 to LUMO+0 HOMO-0 to LUMO+0 transitions occurred. For 4th state, at 868 λ_max_ HOMO-2 to LUMO+0 transition was taking place. Moreover, at λ_max_ 322 (1st state) one transition occurred from HOMO-0 to LUMO+1, while on λ_max_ 305 (5th state) one transition also occurred HOMO-15 to LUMO+0, while for 2nd state at λ_max_ 319 HOMO-4 to LUMO+1 and HOMO-2 to LUMO+1 transitions occurred. For 3rd state at λ_max_ 317 HOMO-2 to LUMO+1 and HOMO-4 to LUMO+0 transitions occurred. For 4th state, at 315 λ_max_ HOMO-13 to LUMO+0 transition was taking place.

### 2.14. NLO Properties

#### 2.14.1. Linear Isotropic and Anisotropic Polarizabilities

As photons travel significantly faster than electrons, optical computers might process data at speeds that are orders of magnitude greater than those of current electronic-based computers if electronic devices were replaced with photonic devices in integrated optics. The above statement triggers us to compute NLO properties for Cd-NDI complex and H_2_-l-ala NDI.

**Figure 17:**
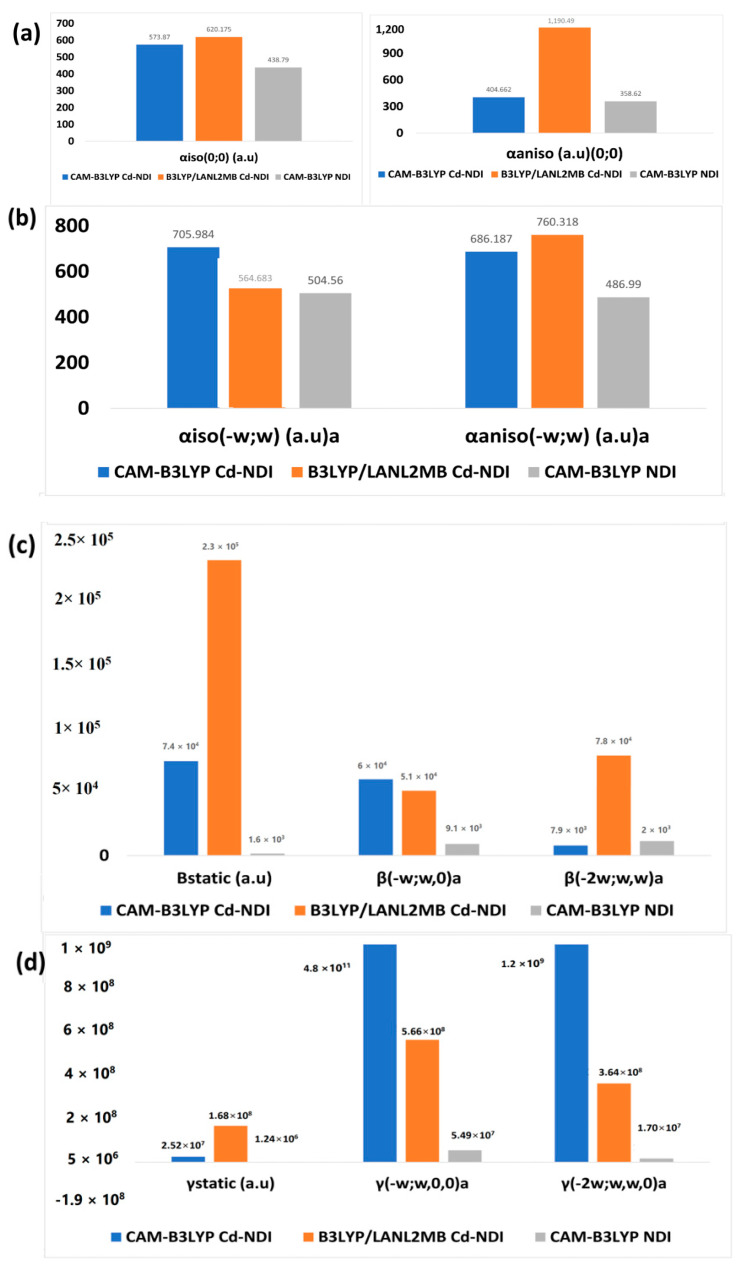
(**a**) Comparisons of linear isotropic (α_iso_) and anisotropic (α_aniso_) of frequency independent; (**b**) frequency dependent (frequency 500.1 nm) (α_iso_) and anisotropic (α_aniso_); (**c**) static first hyperpolarizability β_ο_, electro-optical Pockels (β (−ω; ω; 0)^an^, averages second harmonic generation (β (−2ω, −ω, −ω)^an^; (**d**) static second hyperpolarizability γ_ο_, Kerr effect γ (−ω; ω,0, 0)^an^, second average hyperpolarizability γ (−2ω; ω, ω, 0)^an^ of Cd-NDI complex calculated through CAM-B3LYP/LANL2DZ (**b**) through B3LYP/LANL2MB, and (**c**) H_2_-l-ala NDI through CAM-B3LYP/LANL2DZ, respectively.

Table S10 shows the computed NLO-parameters for the Cd-NDI complex and H_2_-l-ala NDI. The information was gathered using computational sets CAM-B3LYP/LANL2DZ and B3LYP/LANL2MB sets. The Cd-NDI complex and H_2_-l-ala NDI have frequency-independent linear isotropic polarizability (α_iso_) values 573.870 (a.u.) against CAM-B3LYP/LANL2DZ set, 620.175 (a.u.) against B3LYP/LANL2MB set, and 438 (a.u.) against CAM-B3LYP/LANL2DZ, respectively. While the Cd-NDI complex and H_2_-l-ala NDI have frequency-independent linear anisotropic polarizability (α_aniso_) values 404 (a.u.) against CAM-B3LYP/LANL2DZ set, 1190 (a.u.) against B3LYP/LANL2MB set, and 358 (a.u.) against CAM-B3LYP/LANL2DZ, respectively (Figure 17a). The Cd-NDI complex and H_2_-l-ala NDI (Figure 17b) have frequency-dependent linear isotropic polarizability (α_iso_) values 705 (a.u.) against CAM-B3LYP/LANL2DZ set, 464 (a.u.) against B3LYP/LANL2MB set, and 504 (a.u.) against CAM-B3LYP/LANL2DZ, respectively. While the Cd-NDI complex and H_2_-l-ala NDI (Figure 17b) have frequency-dependent linear anisotropic polarizability (α_aniso_) values 686 (a.u.) against CAM-B3LYP/LANL2DZ set, 760 (a.u.) against B3LYP/LANL2MB set, and 486 (a.u.) against CAM-B3LYP/LANL2DZ, respectively.

#### 2.14.2. The First Hyperpolarizability

At a frequency ω = 500.1 nm and frequency independent, the CAM-B3LYP/LANL2DZ and B3LYP/LANL2DMB sets are used to investigate the reliability of the Cd-NDI complex and H_2_-ES-ala NDI (shown in Figure 17c). First hyperpolarizability β_ο_, electro-optical Pockels (β (− ω; ω; 0)^an^, averages second harmonic generation (β (−2ω, − ω, −ω)^an^ were also calculated using both sets. The static first hyperpolarizability β_o_ values of Cd-NDI complex and H_2_-l-ala NDI were found to be 74.5238 and 233,172.0 a.u., respectively. The β_o_ value of the complex was found to be significantly higher than that of its ligand were 745,23 (a.u.), 233,172 (a.u.), and 158,19 (a.u.) using CAM-B3LYP/LANL2DZ and B3LYP/LANL2DMB, respectively. Cd-NDI complex and H_2_-l-ala NDI have electro-optical Pockels (β (−ω; ω; 0)) values of 60,220.4 (using CAM-B3LYP/LANL2DZ set), 511,562.00 (a.u.) using B3LYP/LANL2MB set, 91,556 (a.u.) using CAM-B3LYP/LANL2DZ set, respectively. Cd-NDI complex has average second harmonic generation (β (−2ω: −ω, −ω) of 7966.94, 78,963.80 (a.u.), for both sets, respectively, and H_2_-l-ala NDI have 20,000 (a.u.) [45].

#### 2.14.3. The Second Hyperpolarizability

Computational sets such as CAM-B3LYP/LanL2DZ and B3LYP/LANL2DMB were utilised to explore the second hyperpolarizability of Cd-NDI complex and H_2_-l-ala NDI. The static second hyperpolarizability values for Cd-NDI complex are 252,195,00 and 167,823,000 (a.u.) using CAM-B3LYP/LanL2DZ and B3LYP/LANL2DMB, respectively. Whereas the values for H_2_-l-ala NDI are 1240760 (a.u.) using CAM-B3LYP/LanL2DZ. For Cd-NDI complex and H_2_-l-ala NDI, the γ (−ω; ω, 0, 0) value corresponds to the Kerr effect, which was achieved at 489,038,000,000 (CAM-B3LYP/LanL2DZ) and 565,516,000 (a.u.) (B3LYP/LANL2DMB), 549,295,00 (a.u.) (CAM-B3LYP/LanL2DZ), respectively. The second average hyperpolarizability γ(−2ω; ω, ω, 0) for the Cd-NDI complex is 127,2450,000, and 363,564,000 (a.u.) against CAM-B3LYP/LanL2DZ and B3LYP/LANL2DMB, respectively. While H_2_-l-ala NDI has 169,930,00 using CAM-B3LYP/LanL2DZ set (shown in Figure 17d) [46,47,48,49,50,51].

**Figure 18:**
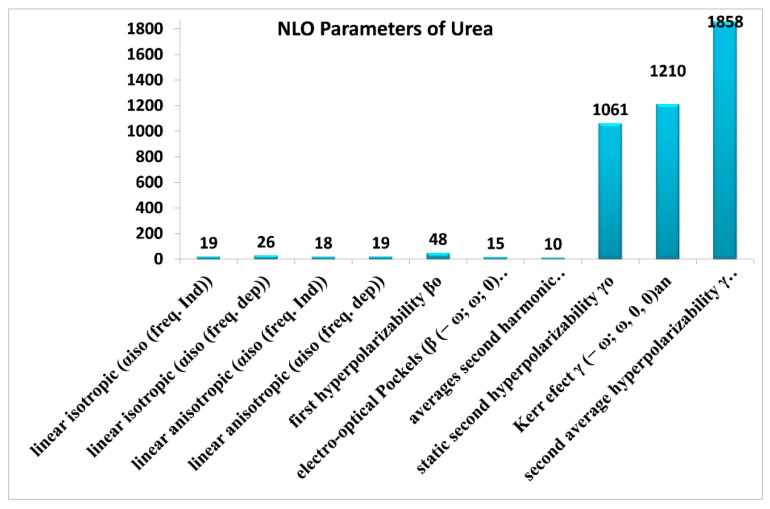
Linear isotropic (α_iso_) and anisotropic (α_aniso_) of frequency independent; frequency dependent (frequency 500.1 nm) (α_iso_) and anisotropic (α_aniso_); static first hyperpolarizability β_ο_, electro-optical Pockels (β (−ω; ω; 0)^an^, averages second harmonic generation (β (−2ω, − ω, −ω)^an^; static second hyperpolarizability γ_ο_, Kerr effect γ (−ω; ω, 0, 0)^an^, second average hyperpolarizability γ (−2ω; ω, ω, 0)^an^ of urea through CAM-B3LYP/LANL2DZ.

**Figure 19:**
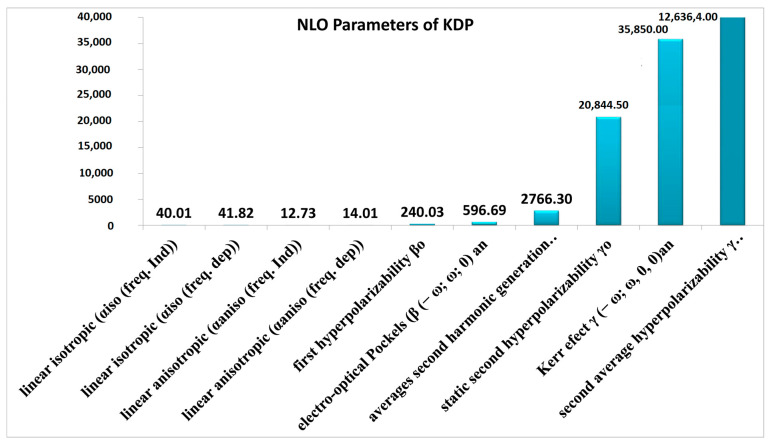
Linear isotropic (α_iso_) and anisotropic (α_aniso_) of frequency independent; frequency dependent (frequency 500.1 nm) (α_iso_) and anisotropic (α_aniso_); static first hyperpolarizability β_ο_, electro-optical Pockels (β (−ω; ω; 0)^an^, averages second harmonic generation (β (−2ω, −ω, −ω)^an^; static second hyperpolarizability γ_ο_, Kerr effect γ (−ω; ω, 0, 0)^an^, second average hyperpolarizability γ (−2ω; ω, ω, 0)^an^ of KDP through CAM-B3LYP/LANL2DZ.

The present work is also compared with already reported work (see Supplementary Materials Table S13). From the above table, it can be seen that averages second harmonic generation or β(−2ω; ω, ω)^a^ values of Cd-NDI complex are 68 esu by using CAM-B3LYP/LANL2DZ set that is much higher than Os complex (43 esu that is experimentally calculated). However, the averages second harmonic generation or β(−2ω; ω, ω)a values of Cd-NDI complex are 682 esu by using B3LYP/LANL2MB set that is much higher than Ni and Cu Schiff bases (235 and 237 esu, experimentally calculated). Furthermore, Cd-NDI complex represents much higher NLO properties than computed properties of not the same, but some similar, complexes (Table S13). Cd-NDI complex is also compared with NDI ligand along with urea (Figure 18) and potassium dihydrogen phosphate (KDP, (Figure 19)) [52]. The results indicate that Cd-NDI complex has much higher NLO properties than urea and KDP. Many researchers have already calculated urea and KDP NLO but we also calculated using CAM-B3LYP/LANL2DZ set to avoid any confusion.

Metal-ligand complex has the benefit over organic systems due to their increased flexibility at the design state, as well as their strong reactions, short response times, integration into composites, and ease of production. Metal-ligand or ligand-metal charge transfer bands can be found in the UV-visible spectrum of the complex (Figure S4).

The inclusion of surplus electrons can significantly improve the NLO performance of a coordinated complex, according to the literature. Surplus electrons occupy high-energy HOMO levels, resulting in a decrease in E_g_ and an increase in β_o_. There is a significant change in the β_o_ of the Cd-NDI complex. Based on the analysis of NLO properties, it is predicted that the Cd-NDI complex will have a spectacular NLO response. The second hyperpolarizability is enhanced by several factors, including flexibility, a long π-delocalization length in π-conjugated, donor and acceptor functional group presence, chain orientation conformation, packing density, and dimensionality.

## 3. Experimental

### 3.1. Materials and Instrumentation

All chemicals that were used in experiments were from a commercial source and were not treated in any way before being utilised. Moreover, Sigma-Aldrich provided naphthalene-1,4,5,8-tetracarboxylic dianhydride (NTDA), L-alanine, Cd(NO_2_)_3_.4H_2_O, and acetic acid. The characterisation of the synthesised material has been designated as an attendant technique. On a Nicolet Nexus FT-IR spectrometer, FT-IR spectra were collected in the 4000–400 cm^−1^ range using KBr pellets. The Persee TU-1950 spectrophotometer was chosen to obtain the UV spectra. For differential scanning calorimetry (DSC) under nitrogen gas at a heating rate of 20 °C/min, a Q100 TA DSC series thermal analyzer was approved. Using a Japan Rigaku D/max γA X-ray diffractometer and graphite-monochromatized Mo Kα radiation (λ = 0.71073Å), powder X-ray diffraction (PXRD) investigations of materials were performed. Supporting information S1 includes a spectrum. All data on crystal structures were collected in the Australian Synchrotron on the MX1 or MX2 beamlines while assassinating at 17.4 keV and using BluIce control software.

### 3.2. Single-Crystal Analysis

#### Crystal Structure Refinement Details of Cd-NDI Complex

At room temperature, hydrocarbon oil was employed to mount the crystal. Graphite monochromator Mo Kα rays with wavelength λ = 0.71073 Å were used to obtain single crystal data of the Cd-NDI complex. After collecting the data, the OLEX2 programme was utilised to solve and refine the complicated structure using the absorption correction approach. The complex’s assembly was discovered utilising a direct method with OLEX2 and enhanced using the SHELEX XL software. All atoms except hydrogen were refined anisotropically and the hydrogen atoms were refined using OLEX2′s default parameters [53]. Tables S1–S9 show the bond angles, bond lengths, and hydrogen bonding, as well as refinement parameters for a single crystal of the Cd-NDI complex.

### 3.3. Computational Details

Crystal explorer was used for investigating Hirsch field surface analysis. Moreover, all computations for the Cd-NDI complex and H_2_-l-ala NDI were performed using the Gaussian 09 simulation program [54]. The CAM-B3LYP/LANL2DZ and B3LYP/LANL2MB basis sets were used to optimize the geometries of the Cd-NDI complex and H_2_-l-ala NDI. Frequency estimations on the coordinates of optimized geometry were also performed to validate the type of computed geometries. From optimized structures, frontier molecular orbitals (FMOs), molecular geometry, and molecular electrostatic potential (MEP) were derived. Time-dependent density functional theory (TD-DFT) and their respective basis sets with CAM-B3LYP functional was used to perform UV-Vis spectral simulations of the Cd-NDI complex and H_2_-l-ala NDI. To prove the thermodynamic stability of Cd-NDI complex and H_2_-l-ala NDI surfaces, vertical energies (E_VI_) are approximated [55]. Multiwfn 3.7 tool [56] was used to measure electron densities, whereas PyMOlyze 1.1 program 32 was used to display DOS data. Electron density difference map (EDDM), dipole moment (µ), and infrared (IR) analyses all employ the same functional. The most common sign of a molecule’s NLO response is hyperpolarizabilities. The α_iso_ and α_aniso_ (linear static polarizability), β_tatic_ (first hyperpolarizability), and γ_o_ (2nd hyperpolarizability) are computed using the CAM-B3LYP/LANL2DZ and B3LYP/LANL2MB basis sets.

### 3.4. Syntheses of Ligand

To the 80–100 mL Acetic acid, L-alanine (2.095 g, 23.5 mmol) and Naphthalene-1,4,5,8-tetracarboxylic dianhydride (NTDA) (3 g, 11.2 mmol) were mixed and kept under reflux for 35 h. After chilling at room temperature, the resultant whitish-brown liquid was reduced to 10–15 mL using the rotary evaporator. Excess deionized water was used to wash the product until ordinary pH was attained, and the product was filtered and dried [35].

### 3.5. Complex Formation

**Scheme 1:**
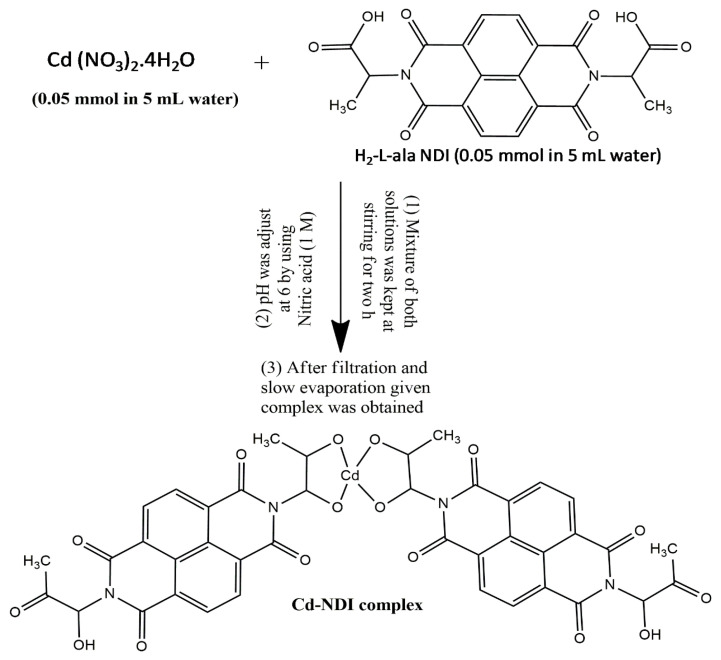
Formation of Cd-NDI complex representing all steps.

Additionally, 12 mg, 0.05 mmol of H_2_-l-ala NDI were dissolved in water (5 mL); similarly, 12 mg, 0.05 mmol of Cd (NO_3_)_2_.4H_2_O were dissolved in 5 mL of water. Next, both solutions were mixed (by stirring for two hours) in a glass vial and pH was adjusted at 6 by using HNO_3_ (1 M) to remove the precipitation (see synthetic Scheme 1 below). The vial was kept for evaporation and needle-like yellowish-red color crystal (ESI Figure S1) was obtained after several days. PXRD spectrum is given in ESI Figure S2. Yield 8.5 mg, (25.5%). IR (Film): υ/cm^−1^ 3450, 2950, 1550, 1460, 1351, 1261, 1085, 1045, 885, 833, 785, 750, 654. TGA: On-set, 25 °C, weight loss = 40% (calculated 40% for loss of H_2_O, CO_2_, and NO_2_).

## 4. Conclusions

The current research is based on a thorough synthesis and analysis of a novel Cd-NDI complex. A simple evaporation process was used to synthesize the Cd-NDI complex. More notably, this is the first time any NDI complex (monomer, crystal) has been produced using water as the solvent. Furthermore, the complex is chiral. Additionally, we used the CAM-B3LYP/LANL2DZ and B3LYP/LANL2MB functionals to investigate NLO characteristics. This study concludes that NLO values are higher against CAM-B3LYP/LANL2DZ basis set. FMOs, DOS, EDDM, TDM, and MEP analyses were used to investigate charge distribution. The thermodynamic stability of complexes has been investigated using *E_VI_*. In comparison to H_2_-l-ala NDI, the simulated results of the Cd-NDI complex are in the range of an efficient NLO material. The newly synthesised Cd-NDI complex has a greater dipole moment (5.36 and 7.86 D) than the H_2_-l-ala NDI (1.14 D). Similarly, the E_g_ of the Cd-NDI complex is 2.62 and 2.42 eV, which is significantly narrower than the E_g_ of the H_2_-l-ala NDI (3.387 eV). To investigate the sorts of vibrations involved, and the nature of the contact, infrared (IR) and NCI analyses were used. Strong metal-ligand coordination and efficient charge transfer resulted in higher electrical conductivities of the Cd-NDI complex. All the computationally estimated parameters support the use of the Cd-NDI complex in integrated NLO devices and point to new avenues for developing highly efficient NLO materials. Furthermore, complications have indicated more virtuous and upstanding consequences. To summarise, this current study provides new insight into the Cd-NDI complex’s tuneable NLO properties. In addition, materials with NLO capabilities could be employed in optical communication, optical computing, optical switching, optical communication, and optical image processing in the future.

## Figures and Tables

**Table 1:** Crystal data and structure refinement for Cd-NDI complex.

Identification Code	Cd-NDI Complex
Empirical formula	C_40_H_28.65_CdN_4_O_17.32_
Formula weight	954.83
*T* (K)	296.15
Crystal system	Orthorhombic
Space group	*P2_1_2_1_2*
a (Å)	24.891(2)
b (Å)	7.3061(7)
c (Å)	11.7772(11)
α (°)	90
β (°)	90
γ (°)	90
*V* (Å^3^)	2141.7(3)
*Z*	2
*ρ*_cald_ (g/cm^3^)	1.481
µ (Mo Kα) (mm^−1^)	0.588
*F*(000)	966.0
Crystal size/mm^3^	0.25 × 0.23 × 0.2
Radiation	Mo Kα (λ = 0.71073)
2Θ range for data collection/°	3.458 to 50.76
Index ranges	−29 ≤ h ≤ 30, −8 ≤ k ≤ 8, −14 ≤ l ≤ 14
Reflections collected	21,339
Unique data (R_int_)	3935 (0.0251)
R_sigma_	0.0206
Data/restraints/parameters	3935/6/290
GOF	1.061
*R*_1_ /w*R*_2_ [*I >* 2*(Iσ)*]	0.0442/0.1273
*R*_1_ /w*R*_2_ [all data]	0.0464/0.1298
maximum/minimum (e/Å^−3^)	2.19/−0.41
Flack parameter	−0.003(8)

**Table 2:** Hydrogen Bonds for Cd-NDI complex.

D	H	A	d(D-H)/Å	d(H-A)/Å	d(D-A)/Å	D-H-A/°
O1	H1	O4 ^1^	0.82	1.80	2.584(6)	158.2 ^1^

^1^ Belongs to first O4, as there are two O4 oxygens in structure.

**Table 3:** Electron-hole analysis of Cd-NDI complex (1–5 excited states) through CAM-B3LYP/LANL2DZ.

Excited State	1	2	3	4	5
E(eV)	1.196 eV	1.340 eV	1.386 eV	1.428 eV	1.526 eV
λ_max_ (nm)	322	319	317	315	305
D(Å)	12.651	7.416	5.090	20.768	10.744
F	0.0394	0.5093	0.4130	0.0075	0.0006
S	0.17161	0.39821	0.41036	0.01619	0.31276
Transition mode	CT	CT	CT	CT	CT

**Table 4:** Electron-hole analysis of Cd-NDI complex (1–5 excited states) through B3LYP/LANL2MB.

Excited State	1	2	3	4	5
E(eV)	3.84	3.882 eV	3.906 eV	3.934 eV	3.946 eV
λ_max_ (nm)	1036.6	925.2	894.3	868.2	853.8
D(Å)	4.251	1.111	1.119	6.158	1.686
F	0.0004	0.0007	0.0015	0.0045	0.0001
S	0.19131	0.53443	0.53190	0.25832	0.39650
Transition mode	CT	CT	CT	CT	CT

## Data Availability

Not applicable.

## Supplementary Materials

The following supporting information can be downloaded at: https://www.mdpi.com/article/10.3390/molecules28093709/s1, Figure S1: Image of Cd-NDI complex crystal.; Figure S2: PXRD of Cd-NDI complex crystal; Figure S3: TGA of Cd-NDI complex crystal; Table S1: Fractional Atomic Coor-dinates (×104) and Equivalent Isotropic Displacement Parameters (Å2×103) for Cd-NDI complex. Ueq is defined as 1/3 of of the trace of the orthogonalised UIJ tensor.; Table S2: Anisotropic Displacement Parameters (Å2×103) for Cd-NDI complex; Table S3: Bond Lengths for Cd-NDI complex; Table S4: Bond Angles for Cd-NDI complex; Table S5: Hydrogen Bonds for Cd-NDI complex; Table S6: Torsion Angles for Cd-NDI complex; Table S7: Hydrogen Atom Coordinates (Å×104) and Isotropic Displacement Parameters (Å2×103) for Cd-NDI complex; Table S8: Atomic Occupancy for Cd-NDI complex; Table S9: Solvent masks information for Cd-NDI complex; Table S10: All DOS infor-mation; Table S11: Electronic transition of Cd-NDI complex through CAM-CAM-B3LYP/LANL2DZ. Table S12: Electronic transition of Cd-NDI complex through B3LYP/LANL2MB; Table S13: Comparison on NLO properties of present work with already reported work; Figure S4: UV-visible absorption graphs of (a) experi-mental Cd-NDI complex, (b) computational of Cd-NDI complex through CAM-B3LYP/LANL2DZ (c) computational of Cd-NDI complex through B3LYP/LANL2MB and (d) computational of H2-L-ala NDI through CAM-CAM-B3LYP/LANL2DZ, respectively; Figure S5: IR spectra of (a) experimental Cd-NDI complex, (b) computational of Cd-NDI complex through CAM-CAM-B3LYP/LANL2DZ (c)) computational of Cd-NDI complex through B3LYP/LANL2MB and (d) computational of H2-L-ala NDI through CAM-CAM-B3LYP/LANL2DZ, respectively; References [57,58,59,60,61] are cited in the Supplementary Materials.

## Sample Availability

Samples of the compounds Cd-NDI complex are available from the authors.
