# Supplementary material for: The Electronic Properties of Cadmium Naphthalene Diimide Coordination Complex
Source: Molecules. 2023 Apr 25;28(9):3709. doi: 10.3390/molecules28093709 (PMC10180404; doi:10.3390/molecules28093709)

checkCIF/PLATON report

You have not supplied any structure factors. As a result the full set of tests cannot be run.

THIS REPORT IS FOR GUIDANCE ONLY. IF USED AS PART OF A REVIEW PROCEDURE FOR PUBLICATION, IT SHOULD NOT REPLACE THE EXPERTISE OF AN EXPERIENCED CRYSTALLOGRAPHIC REFEREE.

No syntax errors found. CIF dictionary Interpreting this report

Datablock: 1_a

Bond precision:	C-C = 0.0061 A		Wavelength=0.71073
Cell:	a=24.891(2)	b=7.3061(7)	c=11.7772(11)
	alpha=90	beta=90	gamma=90
Temperature:	296 K		

	Calculated	Reported
Volume	2141.8(3)	2141.7(3)
Space group	P 21 21 2	P 21 21 2
Hall group	P 2 2ab	P 2 2ab
Moiety formula	C40 H30 Cd N4 O18, O [+ solvent]	C40 H30 Cd N4 O18, O
Sum formula	C40 H30 Cd N4 O19 [+ solvent]	C40 H30 Cd N4 O19
Mr	983.09	983.08
Dx,g cm-3	1.524	1.524
Z	2	2
Mu (mm-1)	0.593	0.593
F000	996.0	996.0
F000’	994.97	
h,k,lmax	30,8,14	30,8,14
Nref	3939[2280]	3935
Tmin,Tmax	0.862,0.888	0.633,0.745
Tmin’	0.862	

Correction method= # Reported T Limits: Tmin=0.633 Tmax=0.745
AbsCorr = MULTI-SCAN

Data completeness= 1.73/1.00 Theta(max)= 25.380

R(reflections)= 0.0305(3748) wR2(reflections)= 0.0822(3935)

S = 1.027 Npar= 304

The following ALERTS were generated. Each ALERT has the format
test-name_ALERT_alert-type_alert-level.
Click on the hyperlinks for more details of the test.

Alert level B

```
PLAT260_ALERT_2_B Large Average Ueq of Residue Including        O9      0.413 Check
PLAT306_ALERT_2_B Isolated Oxygen Atom (H-atoms Missing ?) .......        O9 Check
PLAT420_ALERT_2_B D-H Bond Without Acceptor  O5       --H5B      .     Please Check
```

Alert level C

```
ABSTY02_ALERT_1_C  An _exptl_absorpt_correction_type has been given without
            a literature citation. This should be contained in the
            _exptl_absorpt_process_details field.
            Absorption correction given as multi-scan
PLAT090_ALERT_3_C Poor Data / Parameter Ratio (Zmax > 18) ........       7.50 Note
PLAT094_ALERT_2_C Ratio of Maximum / Minimum Residual Density ....       2.08 Report
PLAT241_ALERT_2_C High   'MainMol' Ueq as Compared to Neighbors of       O7 Check
PLAT767_ALERT_4_C INS Embedded LIST 6 Instruction Should be LIST 4     Please Check
```

Alert level G

```
PLAT007_ALERT_5_G Number of Unrefined Donor-H Atoms .............          5 Report
PLAT300_ALERT_4_G Atom Site Occupancy of O2         Constrained at        0.5 Check
PLAT300_ALERT_4_G Atom Site Occupancy of O5         Constrained at        0.5 Check
PLAT300_ALERT_4_G Atom Site Occupancy of H2A        Constrained at        0.5 Check
PLAT300_ALERT_4_G Atom Site Occupancy of H2B        Constrained at        0.5 Check
PLAT300_ALERT_4_G Atom Site Occupancy of H5A        Constrained at        0.5 Check
PLAT300_ALERT_4_G Atom Site Occupancy of H5B        Constrained at        0.5 Check
PLAT301_ALERT_3_G Main Residue  Disorder ..............(Resd  1  )         3% Note
PLAT304_ALERT_4_G Non-Integer Number of Atoms in ..... (Resd  2  )       0.50 Check
PLAT432_ALERT_2_G Short Inter X...Y Contact  O1       ..C11      .       3.00 Ang.
                                        1/2-x,1/2+y,1-z  =      3_556 Check
PLAT432_ALERT_2_G Short Inter X...Y Contact  O6       ..C1       .       3.01 Ang.
                                        1/2-x,1/2+y,1-z  =      3_556 Check
PLAT605_ALERT_4_G Largest Solvent Accessible VOID in the Structure        131 A**3
PLAT779_ALERT_4_G Suspect or Irrelevant (Bond) Angle(s) in CIF ...       0.00 Deg.
              CD1  -O4   -CD1     2_655   1_555   1_555 ......     #   38 Check
PLAT779_ALERT_4_G Suspect or Irrelevant (Bond) Angle(s) in CIF ...       0.00 Deg.
              CD1  -O7   -CD1     2_655   1_555   1_555 ......     #   53 Check
PLAT779_ALERT_4_G Suspect or Irrelevant (Bond) Angle(s) in CIF ...       0.00 Deg.
              CD1  -O5   -CD1     2_655   1_555   1_555 ......     #  128 Check
PLAT779_ALERT_4_G Suspect or Irrelevant (Bond) Angle(s) in CIF ...      41.30 Deg.
              O2   -O5   -H5B     1_555   1_555   1_555 ......     #  136 Check
PLAT789_ALERT_4_G Atoms with Negative _atom_site_disorder_group  #          3 Check
PLAT791_ALERT_4_G Model has Chirality at C2          (Sohnke SpGr)          S Verify
PLAT791_ALERT_4_G Model has Chirality at C8          (Sohnke SpGr)          S Verify
PLAT868_ALERT_4_G ALERTS Due to the Use of _smtbx_masks Suppressed          ! Info
PLAT883_ALERT_1_G No Info/Value for _atom_sites_solution_primary .     Please Do !
PLAT933_ALERT_2_G Number of HKL-OMIT Records in Embedded .res File          3 Note
```

```
 0 ALERT level A = Most likely a serious problem - resolve or explain
 3 ALERT level B = A potentially serious problem, consider carefully
 5 ALERT level C = Check. Ensure it is not caused by an omission or oversight
22 ALERT level G = General information/check it is not something unexpected

 2 ALERT type 1 CIF construction/syntax error, inconsistent or missing data
 8 ALERT type 2 Indicator that the structure model may be wrong or deficient
 2 ALERT type 3 Indicator that the structure quality may be low
17 ALERT type 4 Improvement, methodology, query or suggestion
 1 ALERT type 5 Informative message, check
```

It is advisable to attempt to resolve as many as possible of the alerts in all categories. Often the minor alerts point to easily fixed oversights, errors and omissions in your CIF or refinement strategy, so attention to these fine details can be worthwhile. In order to resolve some of the more serious problems it may be necessary to carry out additional measurements or structure refinements. However, the purpose of your study may justify the reported deviations and the more serious of these should normally be commented upon in the discussion or experimental section of a paper or in the "special_details" fields of the CIF. checkCIF was carefully designed to identify outliers and unusual parameters, but every test has its limitations and alerts that are not important in a particular case may appear. Conversely, the absence of alerts does not guarantee there are no aspects of the results needing attention. It is up to the individual to critically assess their own results and, if necessary, seek expert advice.

Publication of your CIF in IUCr journals

A basic structural check has been run on your CIF. These basic checks will be run on all CIFs submitted for publication in IUCr journals (*Acta Crystallographica*, *Journal of Applied Crystallography*, *Journal of Synchrotron Radiation*); however, if you intend to submit to *Acta Crystallographica Section C* or *E* or *IUCrData*, you should make sure that full publication checks are run on the final version of your CIF prior to submission.

Publication of your CIF in other journals

Please refer to the *Notes for Authors* of the relevant journal for any special instructions relating to CIF submission.

PLATON version of 28/11/2022; check.def file version of 28/11/2022

Datablock 1_a - ellipsoid plot

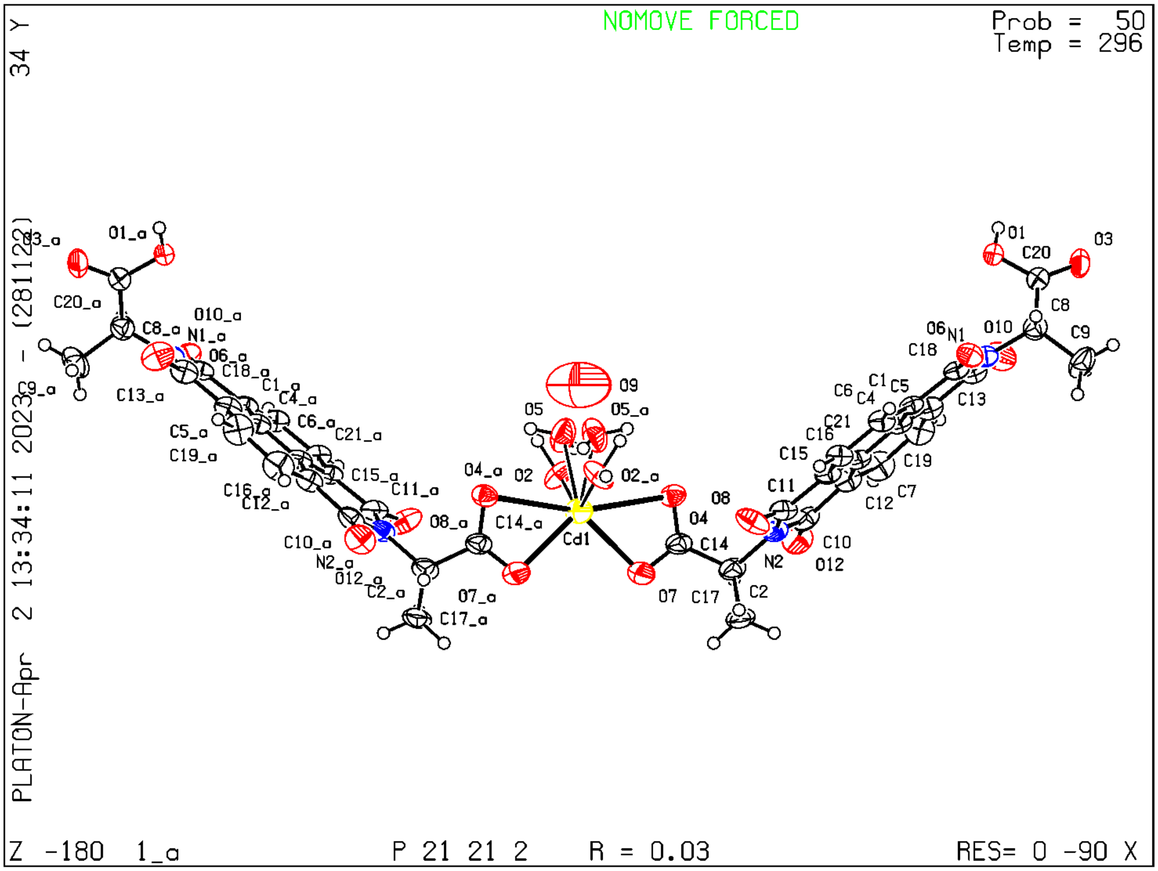

Supplement: Supplementary file 1 [file molecules-28-03709-s001.zip › checkcif.pdf]
