# Supplementary material for: The Electronic Properties of Cadmium Naphthalene Diimide Coordination Complex
Source: Molecules. 2023 Apr 25;28(9):3709. doi: 10.3390/molecules28093709 (PMC10180404; doi:10.3390/molecules28093709)
Supplement: Supplementary file 1 [file molecules-28-03709-s001.zip › molecules-2308228 supplementary materials.pdf]

Supplementary Materials

The Electronic Properties of Cadmium Naphthalene Diimde Coordination Complex

Wajid Hussain, Maroof Ahmad Khan, Zhongkui Li, Muhammad Javed Iqbal, Mubashar Ilyas and Hui Li*

Key Laboratory of Cluster Sciences of Ministry of Education Beijing Key Laboratory of Photoelectronic/ Electro photonic Conversion Materials, School of Chemistry and Chemical Engineering, Beijing Institute of Technology, Beijing 102488, People's Republic of China.

* Correspondence: lihui@bit.edu.cn

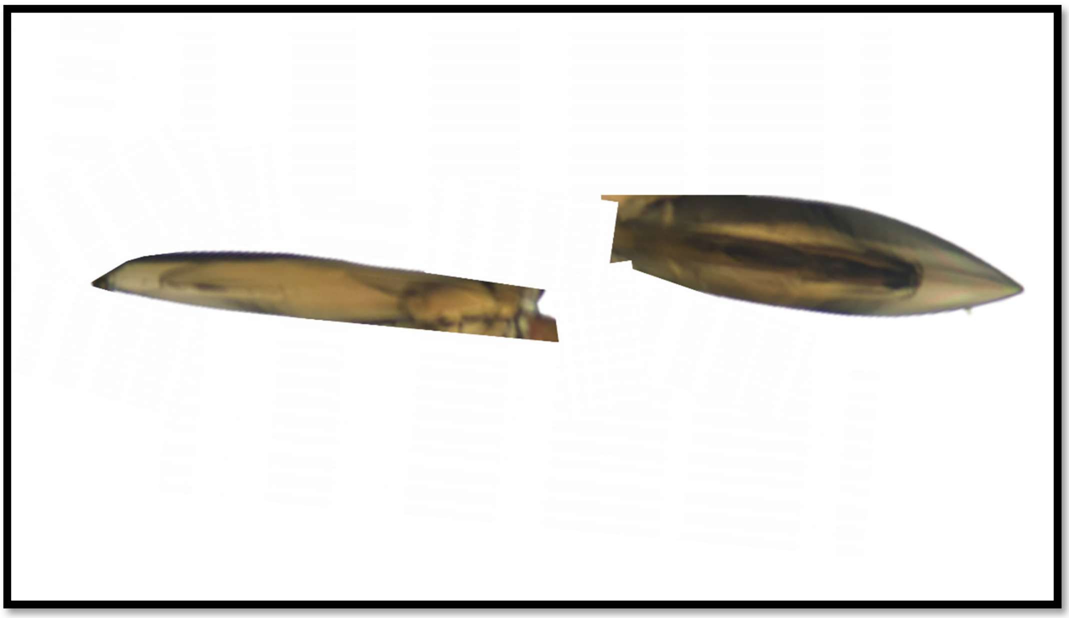

Figure S1: Image of Cd-NDI complex crystal.

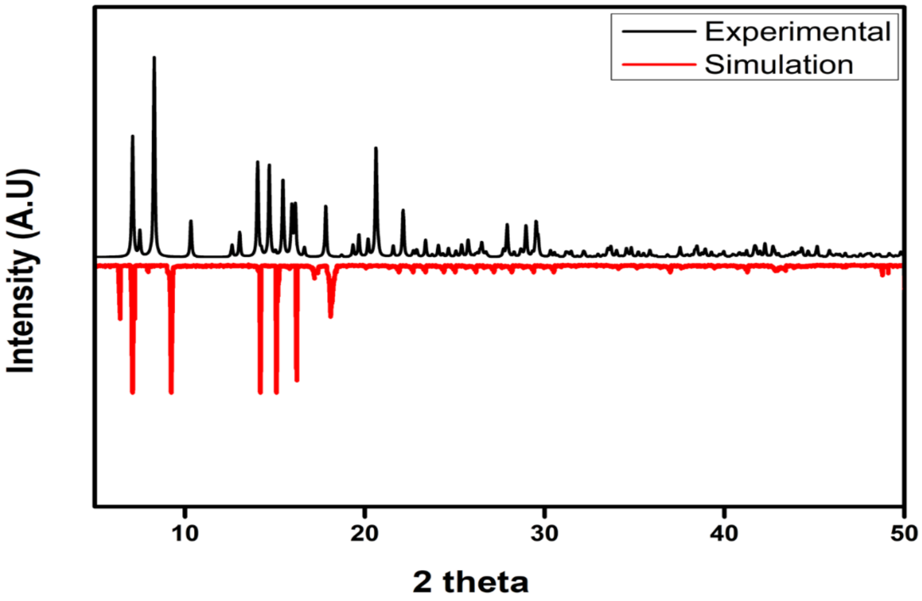

Figure S2: PXRD of Cd-NDI complex crystal.

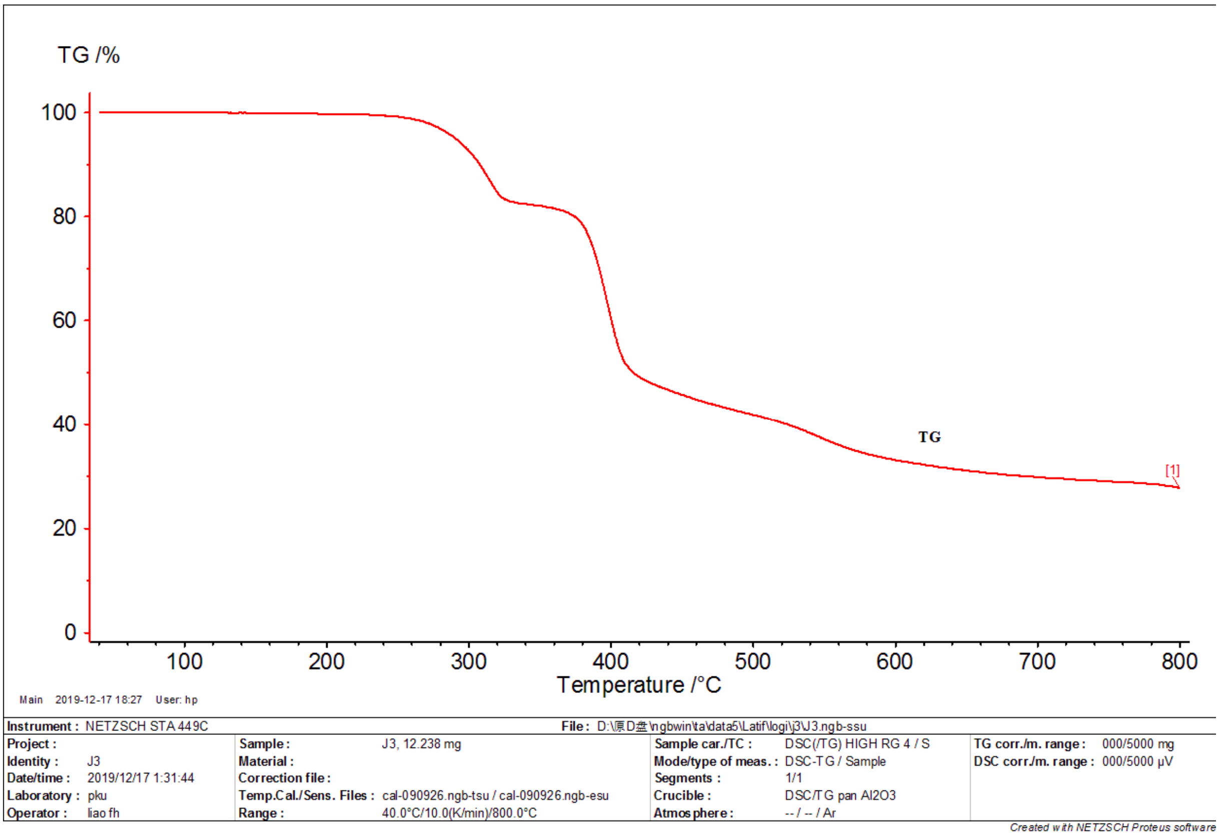

Figure S3: TGA of Cd-NDI complex crystal.

Table S1: Fractional Atomic Coordinates (×10⁴) and Equivalent Isotropic Displacement Parameters (Å²×10³) for *Cd-NDI complex*. U_{eq} is defined as 1/3 of of the trace of the orthogonalised U_{IJ} tensor.

Atom	*x*	*Y*	*z*	U(eq)
Cd1	5000	0	1159.0(5)	43.1(2)
O1	1371.0(17)	7616(6)	6733(4)	38.9(10)
O4	4062.3(16)	1154(6)	1492(3)	37.2(9)
O6	2256.3(19)	10626(6)	4541(4)	37.7(10)
O8	3889(2)	5283(7)	938(4)	54.2(13)
O10	996(2)	6133(7)	4510(5)	55.0(13)
N1	1619(2)	8403(7)	4510(4)	32.9(11)
O12	2531(2)	1205(6)	533(4)	55.7(13)
O3	577.1(19)	9029(8)	6548(5)	56.6(14)
N2	3207(2)	3254(7)	731(4)	38.3(12)
C1	2462(2)	7999(7)	3452(5)	29.2(11)
C4	2271(2)	6301(7)	3056(5)	31.3(12)
O7	4400(3)	872(9)	-195(4)	74.7(19)
C6	2955(3)	8638(7)	3109(5)	33.5(12)
C8	1241(3)	9578(8)	5151(5)	40.1(14)
C10	2699(3)	2591(8)	983(5)	39.8(14)
C12	2384(3)	3625(8)	1818(5)	37.5(13)
C14	4035(3)	1399(9)	449(5)	39.7(14)
C16	2582(2)	5285(8)	2278(4)	32.4(12)
C18	2119(2)	9111(8)	4211(5)	30.1(13)
C20	1023(3)	8684(9)	6197(5)	40.0(13)
C5	1764(3)	5611(8)	3398(6)	38.1(13)
C11	3426(3)	4881(9)	1169(4)	40.2(13)
C3	3273(3)	7612(8)	2359(5)	36.4(13)
C13	1432(3)	6667(8)	4181(5)	38.6(14)
C7	1875(3)	3039(9)	2130(7)	50.8(18)
C15	3091(3)	5972(8)	1950(5)	33.1(12)
C2	3532(3)	2203(9)	-94(5)	44.3(16)
C17	3648(4)	3288(10)	-1178(5)	61(2)
C9	793(3)	10273(11)	4369(7)	57(2)
C19	1571(3)	4021(9)	2947(7)	50.4(17)
O2	4749(4)	-2500(14)	2002(8)	68(4)

Table S2: Anisotropic Displacement Parameters (Å^2×10^3) for *Cd-NDI complex*. The Anisotropic displacement factor exponent takes the form: $-2\pi^2[h^2a^{*2}U_{11}+2hka^*b^*U_{12}+\ldots]$.

Atom	U_{11}	U_{22}	U_{33}	U_{23}	U_{13}	U_{12}
Cd1	27.6(3)	61.9(4)	39.8(3)	0	0	-13.7(3)
O1	33(2)	51(3)	33(2)	4(2)	-1.2(18)	13.5(19)
O4	33(2)	48(2)	31(2)	0.9(18)	-4.3(17)	-8.9(18)
O6	46(2)	30(2)	37(2)	-7.7(17)	0(2)	-3.3(18)
O8	74(3)	48(3)	41(2)	-17(2)	22(2)	-19(3)
O10	53(3)	54(3)	58(3)	2(2)	15(2)	-18(2)
N1	41(3)	33(3)	25(2)	4.6(18)	0(2)	-3(2)
O12	73(3)	33(2)	61(3)	-20(2)	-20(3)	-2(2)
O3	41(3)	71(3)	57(3)	17(3)	8(2)	27(2)
N2	56(3)	28(3)	31(2)	-8(2)	-12(2)	6(2)
C1	40(3)	24(2)	23(2)	4(2)	-5(2)	-1(2)
C4	42(3)	23(2)	28(3)	6(2)	-7(2)	-9(2)
O7	101(5)	90(4)	34(3)	-6(3)	3(3)	48(4)
C6	47(3)	21(2)	32(3)	-4(2)	-3(2)	-11(2)
C8	39(3)	38(4)	43(3)	5(3)	-1(3)	0(2)
C10	55(4)	28(3)	36(3)	1(3)	-17(3)	-1(3)
C12	54(4)	22(3)	36(3)	-3(2)	-10(3)	-5(3)
C14	58(4)	31(3)	30(3)	-6(2)	-1(3)	-1(3)
C16	47(3)	23(3)	28(2)	1(2)	-9(2)	-4(3)
C18	37(3)	32(3)	22(3)	5(2)	-4(2)	-7(2)
C20	42(3)	44(3)	34(3)	5(3)	-3(3)	7(3)
C5	48(3)	31(3)	36(3)	8(2)	-3(3)	-7(2)
C11	61(4)	34(3)	26(2)	0(4)	-1(2)	-3(3)
C3	46(3)	29(3)	34(3)	2(2)	-1(3)	-8(3)
C13	49(4)	30(3)	37(3)	8(2)	-2(3)	-11(3)
C7	64(5)	25(3)	64(4)	-11(3)	-13(4)	-14(3)
C15	50(3)	22(3)	28(3)	-1(2)	-7(2)	-3(2)
C2	70(5)	36(3)	26(3)	-10(2)	-11(3)	7(3)
C17	105(6)	51(4)	27(3)	-8(3)	-14(4)	36(4)
C9	51(4)	54(5)	66(4)	33(4)	-6(3)	6(4)
C19	53(4)	33(3)	65(5)	-2(3)	-3(4)	-15(3)
O2	67(4)	68(4)	69(4)	2.9(13)	-0.5(13)	-1.0(13)

Table S3: Bond Lengths for Cd-NDI complex

Atom	Atom	Length/Å	Atom	Atom	Length/Å
Cd1	Cd1[1]	0.0000(11)	C1	C4	1.408(8)
Cd1	O4[1]	2.512(4)	C1	C6	1.373(8)
Cd1	O4	2.512(4)	C1	C18	1.479(9)
Cd1	O7	2.276(6)	C4	C16	1.410(8)
Cd1	O7[1]	2.276(6)	C4	C5	1.417(9)
Cd1	C14[1]	2.740(7)	O7	C14	1.244(9)
Cd1	O2[1]	2.171(10)	C6	C3	1.403(9)
Cd1	O2	2.171(10)	C8	C20	1.497(9)
O1	C20	1.327(7)	C8	C9	1.531(9)
O4	C14	1.242(8)	C10	C12	1.466(9)
O6	C18	1.221(7)	C12	C16	1.417(8)
O8	C11	1.220(8)	C12	C7	1.387(10)
O10	C13	1.217(8)	C14	C2	1.524(10)
N1	C8	1.481(8)	C16	C15	1.417(9)
N1	C18	1.394(8)	C5	C13	1.458(10)
N1	C13	1.405(8)	C5	C19	1.365(9)
O12	C10	1.217(7)	C11	C15	1.475(9)
O3	C20	1.209(8)	C3	C15	1.368(8)
N2	C10	1.387(9)	C7	C19	1.419(11)
N2	C11	1.406(8)	C2	C17	1.530(10)
N2	C2	1.480(8)			

[1]1-X,-Y,+Z

Table S4: Bond Angles for Cd-NDI complex.

Atom	Atom	Atom	Angle/°	Atom	Atom	Atom	Angle/°
Cd1^{1}	Cd1	O4	0(10)	C14	O7	Cd1	97.9(4)
Cd1^{1}	Cd1	O4^{1}	0(10)	C14	O7	Cd1^{1}	97.9(4)
Cd1^{1}	Cd1	O7	0(10)	C1	C6	C3	120.5(5)
Cd1^{1}	Cd1	O7^{1}	0(10)	N1	C8	C20	113.4(5)
Cd1^{1}	Cd1	C14^{1}	0(10)	N1	C8	C9	110.4(5)
Cd1^{1}	Cd1	O2^{1}	0(10)	C20	C8	C9	112.1(5)
Cd1^{1}	Cd1	O2	0(10)	O12	C10	N2	120.7(6)
O4	Cd1	O4^{1}	162.06(19)	O12	C10	C12	122.5(7)
O4	Cd1	C14^{1}	170.67(16)	N2	C10	C12	116.8(5)
O4^{1}	Cd1	C14^{1}	26.92(16)	C16	C12	C10	120.8(6)
O7	Cd1	O4^{1}	144.39(17)	C7	C12	C10	120.4(6)
O7	Cd1	O4	53.52(17)	C7	C12	C16	118.7(6)
O7^{1}	Cd1	O4	144.39(17)	O4	C14	O7	121.2(6)
O7^{1}	Cd1	O4^{1}	53.52(17)	O4	C14	C2	121.0(6)
O7^{1}	Cd1	O7	91.1(3)	O7	C14	C2	117.6(6)
O7	Cd1	C14^{1}	117.77(19)	C4	C16	C12	120.6(5)
O7^{1}	Cd1	C14^{1}	26.71(19)	C4	C16	C15	118.7(5)
O2^{1}	Cd1	O4^{1}	86.8(3)	C15	C16	C12	120.7(6)
O2^{1}	Cd1	O4	85.0(3)	O6	C18	N1	120.4(6)
O2	Cd1	O4^{1}	85.0(3)	O6	C18	C1	122.0(6)
O2	Cd1	O4	86.8(3)	N1	C18	C1	117.6(5)
O2^{1}	Cd1	O7	105.9(3)	O1	C20	C8	114.3(5)
O2	Cd1	O7^{1}	105.9(3)	O3	C20	O1	124.0(6)
O2^{1}	Cd1	O7^{1}	111.5(3)	O3	C20	C8	121.5(6)
O2	Cd1	O7	111.5(3)	C4	C5	C13	119.7(5)
O2^{1}	Cd1	C14^{1}	101.6(3)	C19	C5	C4	120.4(7)
O2	Cd1	C14^{1}	94.5(3)	C19	C5	C13	119.8(6)
O2^{1}	Cd1	O2	125.5(5)	O8	C11	N2	119.2(6)
Cd1^{1}	O4	Cd1	0.00(3)	O8	C11	C15	122.9(6)
C14	O4	Cd1^{1}	86.8(4)	N2	C11	C15	117.8(5)
C14	O4	Cd1	86.8(4)	C15	C3	C6	120.1(6)
C18	N1	C8	118.8(5)	O10	C13	N1	119.7(6)
C18	N1	C13	124.1(5)	O10	C13	C5	122.5(6)
C13	N1	C8	117.0(5)	N1	C13	C5	117.7(5)
C10	N2	C11	124.8(5)	C12	C7	C19	120.7(6)
C10	N2	C2	117.2(5)	C16	C15	C11	118.9(5)
C11	N2	C2	117.9(6)	C3	C15	C16	120.7(6)
C4	C1	C18	119.3(5)	C3	C15	C11	120.4(6)
C6	C1	C4	120.2(5)	N2	C2	C14	111.9(5)
C6	C1	C18	120.4(5)	N2	C2	C17	112.5(5)

Atom	Atom	Atom	Angle/˚	Atom	Atom	Atom	Angle/˚
C1	C4	C16	119.6(5)	C14	C2	C17	113.2(7)
C1	C4	C5	121.3(6)	C5	C19	C7	120.4(7)
C16	C4	C5	119.1(5)	Cd1[1]	O2	Cd1	0.00(3)
Cd1[1]	O7	Cd1	0.00(2)				

[1]1-X,-Y,+Z

Table S5: Hydrogen Bonds for Cd-NDI complex.

D	H	A	d(D-H)/Å	d(H-A)/Å	d(D-A)/Å	D-H-A/°
O1	H1	O4[1]	0.82	1.80	2.584(6)	158.2

[1]1/2-X,1/2+Y,1-Z

Table S6: Torsion Angles for Cd-NDI complex.

A	B	C	D	Angle/˚	A	B	C	D	Angle/˚
Cd1[1]	O4	C14	O7	6.7(7)	C8	N1	C13	O10	-6.4(8)
Cd1	O4	C14	O7	6.7(7)	C8	N1	C13	C5	170.4(5)
Cd1	O4	C14	C2	-178.8(6)	C10	N2	C11	O8	176.0(6)
Cd1[1]	O4	C14	C2	-178.8(6)	C10	N2	C11	C15	-0.3(8)
Cd1	O7	C14	O4	-7.4(8)	C10	N2	C2	C14	-115.4(6)
Cd1[1]	O7	C14	O4	-7.4(8)	C10	N2	C2	C17	115.8(7)
Cd1	O7	C14	C2	177.9(5)	C10	C12	C16	C4	178.2(5)
Cd1[1]	O7	C14	C2	177.9(5)	C10	C12	C16	C15	-0.6(8)
O4	C14	C2	N2	22.1(9)	C10	C12	C7	C19	179.2(6)
O4	C14	C2	C17	150.5(6)	C12	C16	C15	C11	-3.3(8)
O8	C11	C15	C16	-172.4(6)	C12	C16	C15	C3	177.3(6)
O8	C11	C15	C3	7.0(9)	C12	C7	C19	C5	3.2(11)
N1	C8	C20	O1	35.9(8)	C16	C4	C5	C13	-178.5(5)
N1	C8	C20	O3	-148.9(6)	C16	C4	C5	C19	-2.6(9)
O12	C10	C12	C16	-176.6(6)	C16	C12	C7	C19	-3.8(10)
O12	C10	C12	C7	0.3(9)	C18	N1	C8	C20	-128.7(6)
N2	C10	C12	C16	4.1(8)	C18	N1	C8	C9	104.6(6)
N2	C10	C12	C7	-179.0(6)	C18	N1	C13	O10	177.7(6)
N2	C11	C15	C16	3.8(8)	C18	N1	C13	C5	-5.5(8)
N2	C11	C15	C3	-176.8(5)	C18	C1	C4	C16	176.2(5)
C1	C4	C16	C12	-176.3(5)	C18	C1	C4	C5	-2.0(8)
C1	C4	C16	C15	2.5(8)	C18	C1	C6	C3	-177.9(5)
C1	C4	C5	C13	-0.3(9)	C5	C4	C16	C12	1.9(8)
C1	C4	C5	C19	175.7(6)	C5	C4	C16	C15	-179.2(5)

A	B	C	D	Angle/°	A	B	C	D	Angle/°
C1	C6	C3	C15	0.9(9)	C11	N2	C10	O12	177.1(6)
C4	C1	C6	C3	0.1(8)	C11	N2	C10	C12	-3.6(8)
C4	C1	C18	O6	-176.9(5)	C11	N2	C2	C14	66.9(7)
C4	C1	C18	N1	0.6(7)	C11	N2	C2	C17	-61.9(8)
C4	C16	C15	C11	177.8(5)	C13	N1	C8	C20	55.2(7)
C4	C16	C15	C3	-1.5(8)	C13	N1	C8	C9	-71.5(6)
C4	C5	C13	O10	-179.4(6)	C13	N1	C18	O6	-179.1(5)
C4	C5	C13	N1	3.9(9)	C13	N1	C18	C1	3.3(8)
C4	C5	C19	C7	0.1(10)	C13	C5	C19	C7	176.0(7)
O7	C14	C2	N2	-163.2(6)	C7	C12	C16	C4	1.2(8)
O7	C14	C2	C17	-34.8(9)	C7	C12	C16	C15	-177.6(6)
C6	C1	C4	C16	-1.9(8)	C2	N2	C10	O12	-0.4(8)
C6	C1	C4	C5	179.9(6)	C2	N2	C10	C12	178.9(5)
C6	C1	C18	O6	1.2(8)	C2	N2	C11	O8	-6.5(8)
C6	C1	C18	N1	178.7(5)	C2	N2	C11	C15	177.2(5)
C6	C3	C15	C16	-0.2(9)	C9	C8	C20	O1	161.7(6)
C6	C3	C15	C11	-179.5(5)	C9	C8	C20	O3	-23.1(9)
C8	N1	C18	O6	5.0(8)	C19	C5	C13	O10	4.6(10)
C8	N1	C18	C1	-172.6(5)	C19	C5	C13	N1	-172.1(6)

[1]1-X,-Y,+Z

Table S7: Hydrogen Atom Coordinates (Å×10^4) and Isotropic Displacement Parameters (Å^2×10^3) for *Cd-NDI complex.*

Atom	*x*	*y*	*z*	U(eq)
H1	1260.98	7399.98	7375.42	58
H6	3078.56	9759.08	3375.17	40
H8	1443.84	10654.2	5400.63	48
H3	3608.29	8047.17	2139.21	44
H7	1731.54	1990.65	1800.34	61
H2	3310.32	1157.95	-324.66	53
H17A	3315.77	3667.37	-1516.77	92
H17B	3842.68	2530.51	-1702.01	92
H17C	3859.59	4346.56	-993.95	92
H9A	944.88	10644.42	3656.17	86
H9B	617.31	11298.79	4718.14	86
H9C	536.83	9312.65	4244.01	86
H19	1237.88	3579.13	3176.62	60

Atom	*x*	*y*	*z*	U(eq)
H2A	4489.55	-3569.2	1993.55	102
H2B	4791.3	-3059.44	2779.3	102

Table S8: Atomic Occupancy for *Cd-NDI complex*.

Atom	*Occupancy*	Atom	*Occupancy*	Atom	*Occupancy*
O2	0.64(2)	H2A	0.64(2)	H2B	0.64(2)

Table S9: Solvent masks information for *Cd-NDI complex*.

Number	X	Y	Z	Volume	Electron count	Content
1	0.000	-0.593	0.264	168.6	44.2	4.4 water
2	0.500	0.003	0.736	168.6	44.5	4.4 water

Experimental

Single crystals of $C_{40}H_{28.65}CdN_4O_{17.32}$ *Cd-NDI complex* were yellow-redish in color. A suitable crystal was selected and mount on a Bruker APEX-II CCD diffractometer. The crystal was kept at 296.15 K during data collection. Using Olex2 [1], the structure was solved with the Unknown [2] structure solution program using Unknown and refined with the Unknown [3] refinement package using Unknown minimisation.

Crystal structure determination of *Cd-NDI complex*

Crystal Data for $C_{40}H_{28.645}CdN_4O_{17.32}$ (M =954.83 g/mol): orthorhombic, space group $P2_12_12$ (no. 18), a = 24.891(2) Å, b = 7.3061(7) Å, c = 11.7772(11) Å, V = 2141.7(3) Å^3, Z = 2, T = 296.15 K, μ(MoKα) = 0.588 mm^{-1}, $Dcalc$ = 1.481 g/cm^3, 21339 reflections measured (3.458° ≤ 2Θ ≤ 50.76°), 3935 unique (R_{int} = 0.0251, R_{sigma} = 0.0206) which were used in all calculations. The final R_1 was 0.0442 (I > 2σ(I)) and wR_2 was 0.1298 (all data).

Refinement model description

Number of restraints - 6, number of constraints - unknown.

Details:

1. Uiso/Uaniso restraints and constraints

Uanis(O2) ≈ Ueq: with sigma of 0.001 and sigma for terminal atoms of 0.002

2. Others

Sof(O2)=Sof(H13)=Sof(H7)=FVAR(1)

3.a Rotating group:

O2(H13,H7)

3.b Ternary CH refined with riding coordinates:

C8(H6), C2(H12)

3.c Aromatic/amide H refined with riding coordinates:

C6(H4), C3(H8), C7(H10), C19(H3)

3.d Idealised Me refined as rotating group:

C17(H14,H15,H2), C9(H9,H5,H11)

3.e Idealised tetrahedral OH refined as rotating group:

O1(H1)

This report has been created with Olex2, compiled on 2020.11.12 svn.r5f609507 for OlexSys. Please let us know if there are any errors or if you would like to have additional features.

Table S10: All DOS information

Column1	Column2	Column3	Column4	Column5	Column6	Column7
**MO**	**Energy(eV)**	**Energy(eV)**	**Sym. 0**	**Sym.1**	**Sym. 2**	**Sym. 3**
1		-515.214	0	100.1	0	-0.1
2		-515.045	100	0	0	0
3		-514.989	0	100.1	0	-0.1
4		-514.88	0	100.1	0	-0.1
5		-514.866	0	100.1	0	-0.1
6		-514.808	100.1	0	-0.1	0
7		-514.773	100.1	0	-0.1	0
8		-514.77	100.1	0	-0.1	0
9		-514.743	1.4	98.6	0	0
10		-514.741	100.1	0	-0.1	0
11		-514.73	98.6	1.4	0	0
12		-513.686	0	100	0	0
13		-513.574	100.1	0	-0.1	0
14		-512.762	0	100.1	0	-0.1
15		-512.704	0.2	99.9	0	-0.1
16		-512.628	99.3	0.8	-0.1	0
17		-512.619	99.9	0.2	-0.1	0
18		-512.608	0.8	99.3	0	-0.1
19		-387.087	0	100.1	0	-0.1
20		-386.951	0	100.1	0	-0.1

21		-386.897	100.1	0	-0.1	0
22		-386.619	100.1	0	-0.1	0
23		-276.469	0	-0.1	0	100.1
24		-276.445	0	-0.1	0	100.1
25		-276.344	-0.1	0	100.1	0
26		-276.14	0	-0.1	0	100.1
27		-276.055	0	-0.1	0	100.1
28		-275.981	-0.1	0	100.1	0
29		-275.973	-0.1	0	100.1	0
30		-275.955	-0.1	0	100.1	0
31		-274.868	-0.1	0	100.1	0
32		-274.333	0	-0.1	0	100.1
33		-274.32	-0.1	0	100.1	0
34		-274.318	0	-0.1	0	100.1
35		-274.315	0	0	0	100
36		-274.309	-0.1	0	100.1	0
37		-274.286	0	0	0	100
38		-274.205	0	0	0	100
39		-274.199	0	0	100	0
40		-274.176	0	0	100	0
41		-274.158	0	0	0	100
42		-274.148	0	0	0	100
43		-274.145	0	0	0	100
44		-274.133	0	-0.1	0	100.1
45		-274.097	-0.1	0	95.3	4.8
46		-274.081	0	-0.1	4.9	95.2
47		-274.077	0	0	99.9	0.1
48		-274.054	0	0	100	0
49		-274.002	0	0	100	0
50		-273.979	0	0	100	0
51		-273.904	0	-0.1	0	100.1
52		-273.897	0	-0.1	0	100.1
53		-273.873	0	-0.1	0	100.1
54		-273.854	0	-0.1	0	100.1
55		-273.825	-0.1	0	100.1	0
56		-273.783	-0.1	0	100.1	0
57		-273.741	-0.1	0	100.1	0
58		-273.716	-0.1	0	100.1	0
59		-272.971	0	-0.2	0	100.2
60		-272.794	-0.2	0	100.2	0
61		-272.746	0	-0.2	0	100.2
62		-272.729	-0.2	0	100.2	0
63		-28.057	0	69.1	0	30.9

64		-27.834	0	68.1	0	31.9
65		-27.795	67.8	0	32.2	0
66		-27.779	68.1	0.1	31.8	0
67		-27.617	0	70.9	0	29.1
68		-27.476	0	71.3	0	28.7
69		-27.433	72.4	0	27.6	0
70		-27.393	71.2	0.1	28.7	0
71		-27.346	73.1	0	26.9	0
72		-26.633	0	72.5	0	27.5
73		-26.359	19.2	48.8	10.5	21.6
74		-26.286	48.5	19.5	22.9	9.1
75		-25.992	5.6	91.3	1.1	2
76		-25.922	82.6	14.9	2.4	0.1
77		-25.53	82.3	0	17.7	0
78		-24.732	0	81.2	0	18.8
79		-24.442	0	59.1	0	40.9
80		-24.286	0.1	64.3	0.1	35.5
81		-24.261	54.9	0.2	44.9	0
82		-24.134	65.8	0	34.1	0
83		-23.782	0.4	36.8	0.6	62.2
84		-23.72	64.6	0.6	33.3	1.5
85		-23.674	0.9	60.6	1.5	37
86		-23.599	33.3	0.1	66.6	0
87		-22.453	0	5.4	0	94.6
88		-22.343	5.3	0	94.7	0
89		-21.63	0	8.4	0	91.6
90		-21.522	8.2	0	91.8	0
91		-20.34	0	11.2	0	88.8
92		-20.244	12	0	88	0
93		-20.186	0	8	0	92
94		-20.059	7.5	0	92.5	0
95		-19.982	0	21.4	0	78.5
96		-19.905	20.7	0	79.3	0
97		-19.736	0.1	25.2	0.3	74.5
98		-19.671	25.2	0.1	74.6	0.2
99		-18.086	0	18.7	0	81.2
100		-17.928	17.6	0	82.3	0
101		-17.785	0	22.5	0.1	77.5
102		-17.673	22.6	0	77.4	0
103		-17.07	0.1	26.2	2.8	71
104		-17.03	27.8	0.1	72	0.1
105		-16.767	0.3	14.1	34.9	50.8
106		-16.731	2.7	14.9	56.5	25.9

107		-16.719	2	20	45.1	32.9
108		-16.682	0.5	12.5	51.3	35.7
109		-16.651	2.8	1.7	93.6	2
110		-16.624	14.2	0.7	84	1.1
111		-16.588	2.8	1	94.9	1.3
112		-16.551	2.9	0.3	96.5	0.2
113		-16.526	33.7	0.1	66.2	0
114		-16.158	1.7	23.7	8.7	65.8
115		-16.128	22.2	1.9	71	4.9
116		-16.054	0.1	30.7	1.8	67.4
117		-15.901	31.6	0.1	68.4	0
118		-14.769	0	28.9	0.1	71
119		-14.588	24.6	0	75.3	0
120		-14.568	0.1	23.8	0.7	75.4
121		-14.468	25.2	0.2	74.5	0.1
122		-14.015	0	30.7	0.2	69.1
123		-13.937	32.5	0.2	67.2	0.1
124		-13.704	0.5	20.7	1.1	77.7
125		-13.664	31.4	0.6	66.9	1.1
126		-13.48	1.1	62.1	1	35.7
127		-13.409	50.6	0.9	48.3	0.3
128		-13.24	52.7	11.5	31.3	4.5
129		-13.174	5.7	49.4	3.1	41.9
130		-12.945	1.5	46.8	2	49.7
131		-12.894	38.6	4.5	55.3	1.6
132		-12.611	0.1	32.9	0.1	66.9
133		-12.533	1	37.1	2.9	59
134		-12.507	35.2	2.2	61	1.6
135		-12.499	34.8	0.5	64.3	0.4
136		-12.207	16.3	0.6	82.5	0.5
137		-12.165	0.2	24.3	0.7	74.8
138		-11.814	0.1	39.3	0.2	60.5
139		-11.752	40.5	0.2	59.3	0.1
140		-11.697	0.4	34.6	0.9	64.1
141		-11.674	33.8	1.2	64.2	0.9
142		-11.492	0	25.7	0.2	74
143		-11.373	24.5	0.3	75.2	0.1
144		-11.36	0	16.8	0.1	83.1
145		-11.25	3.2	32.9	15.2	48.8
146		-11.246	13.2	7.6	68.2	11
147		-11.162	39.6	0.2	60.1	0
148		-10.88	0.7	55.7	1.5	42.1
149		-10.808	0.5	33.7	0.7	65.1

150		-10.749	39.2	1.6	58	1.3
151		-10.659	42	3	53.9	1
152		-10.64	0.6	30.8	1.2	67.5
153		-10.54	29.4	6.7	57	6.8
154		-10.511	9.1	41.7	16.8	32.4
155		-10.497	9.9	23.7	22.3	44.1
156		-10.479	23	14.5	38.9	23.7
157		-10.396	36.9	3.1	57.8	2.2
158		-10.359	1.1	47.8	1.5	49.7
159		-10.27	18.7	36	11.8	33.5
160		-10.212	42.9	22.2	21.7	13.2
161		-10.052	50	0.9	48.8	0.3
162		-9.964	0.1	53.4	0.1	46.5
163		-9.89	5.3	38.3	4.1	52.4
164		-9.829	46.3	6.9	35.8	10.9
165		-9.787	13.6	24.5	19.4	42.6
166		-9.773	31.4	6.7	49.5	12.4
167		-9.729	5.5	43.5	9.3	41.7
168		-9.711	30.4	6.4	54.4	8.8
169		-9.688	42.8	2.7	52.4	2.2
170		-9.649	1.5	42.2	2.3	54
171		-9.534	38	16	28.9	17.1
172		-9.513	18.7	30.6	14.1	36.7
173		-9.472	43.8	1.2	53.6	1.4
174		-9.334	41.8	7.5	44.6	6.1
175		-9.301	13.1	31.7	19.4	35.8
176		-9.273	0.4	40.9	0.4	58.3
177		-9.232	22.8	27.9	26.4	22.9
178		-9.148	19.1	24.1	28.9	27.9
179		-9.112	16.4	24.5	22.5	36.6
180		-9.01	41.8	3.8	50.7	3.7
181		-8.928	4.3	43.3	4.4	48
182		-8.891	38.5	11.8	34.3	15.4
183		-8.834	23.4	22.7	31.7	22.2
184		-8.766	30.1	15.9	38.9	15.1
185		-8.625	71.6	0.8	27	0.6
186		-8.486	34.9	23.2	29.6	12.3
187		-8.372	54.9	0.1	45	0
188		-8.189	3.7	48.3	2.5	45.5
189		-8.132	7.1	54.3	6.1	32.5
190		-8.065	16	38.8	21.9	23.2
191		-8.029	11.4	37.6	17	34
192		-8.005	22.7	38.7	16.1	22.5

193		-7.906	30.1	16.5	42.7	10.7
194		-7.748	14.1	42.5	9.8	33.6
195		-7.624	47.2	19.4	29.3	4.1
196		-7.378	77.1	0	22.8	0
197		-7.207	0.5	21.4	0.8	77.3
198		-7.15	0.1	78.8	0.4	20.7
199		-7.082	21.6	1.2	76.5	0.8
200		-6.51	0	0.3	0	99.6
201		-6.404	0.5	0	99.5	0
202		-6.112	23.4	53.4	18	5.1
203		-5.717	88	0.8	11	0.1
204		-5.71	0.8	89.9	0.1	9.2
205		-5.617	24.6	60.7	3.6	11.1
206		-5.578	18.2	69.9	2.2	9.7
207		-5.553	76.7	6.3	13.4	3.6
208	-19	-5.495	25.8	26.1	6.1	42
209	-18	-5.444	20.7	44.2	9.6	25.5
210	-17	-5.401	69.7	16.6	10.3	3.3
211	-16	-5.358	46.1	3.3	50.4	0.2
212	-15	-5.342	0.4	88.4	0.2	11
213	-14	-5.317	78.4	0.7	20.8	0
214	-13	-5.127	0.2	91.4	0	8.5
215	-12	-5.049	90.8	1.2	7.9	0.1
216	-11	-4.959	0.1	92.9	0	7.1
217	-10	-4.935	93.6	0.6	5.8	0.1
218	-9	-4.918	95.4	0.4	4.2	0
219	-8	-4.861	30	49.1	16.2	4.7
220	-7	-4.181	40.6	36.3	16.1	6.9
221	-6	-4.109	0	98.7	0	1.3
222	-5	-3.995	89	0	11	0
223	-4	-3.508	52.2	47.1	0.6	0.1
224	-3	-3.485	47	52.8	0.2	0
225	-2	-3.429	0	87.7	0	12.3
226	-1	-3.304	53.6	45.3	0.9	0.2
227	0	-3.207	45.9	53	1	0.2
**---**	**HOMO**	**- LUMO gap ---**	**1.6**	**56 eV -**	**-------**	**-------**
228	0	-1.551	0	28.3	0	71.6
229	1	-1.392	27.6	0	72.3	0
230	2	0.362	0	29.9	0.2	69.8
231	3	0.485	29.2	0	70.7	0
232	4	0.722	0	20.6	0.1	79.3
233	5	0.725	0	14.3	0.1	85.7
234	6	0.825	0.7	0	99.3	0

235	7	0.881	35.2	0.1	64.7	0
236	8	2.04	0.2	32.1	1.7	66
237	9	2.199	37.9	0.3	61.8	0.1
238	10	2.327	6.1	25.2	18.3	50.4
239	11	2.401	15.3	17.1	47.6	20.1
240	12	2.46	22.7	8.4	65.2	3.7
241	13	2.826	50.1	0	49.9	0
242	14	3.022	14.5	16.6	50.4	18.6
243	15	3.241	0	50.3	0	49.7
244	16	3.32	18.8	17.7	45.7	17.8
245	17	4.018	9.1	23.2	49.3	18.4
246	18	4.186	10.5	8	25.4	56.1
247	19	4.28	7.9	11.9	37.4	42.8
248		4.379	6.5	3.3	79.3	10.8
249		4.436	2.3	19.6	14.5	63.6
250		4.538	23.2	4.3	70.3	2.2
251		5.916	0	29.5	0	70.5
252		5.919	30.2	0	69.8	0
253		6.123	0.3	29.1	1.7	68.9
254		6.257	24.9	3.4	70.3	1.5
255		6.474	14.8	15.3	63.1	6.7
256		6.977	0	3.8	0.1	96.2
257		7.079	3.8	0.1	95.8	0.2
258		7.205	2.7	19.7	9.6	68
259		7.243	21.3	3	67.4	8.3
260		7.593	30.3	0.4	69	0.3
261		7.691	0.1	25.1	0.2	74.7
262		7.899	35	0.4	64.2	0.4
263		7.92	0	43.1	0	56.9
264		8.075	1.2	40.4	2.9	55.5
265		8.117	55.2	0.5	43.8	0.5
266		8.234	45.8	0.4	53.1	0.7
267		8.362	0	16.1	0.1	83.8
268		8.492	14.9	0.4	83.5	1.1
269		8.505	0.3	25.1	1.7	72.9
270		8.637	54.3	0.1	45.4	0.2
271		8.705	0.1	41.2	0.1	58.6
272		8.748	0	33.3	0.1	66.6
273		8.783	39.5	0	60.4	0
274		8.878	35.8	0.2	63.9	0.1
275		9.413	24.1	6.5	61.3	8.1
276		9.439	4.6	35.3	4.5	55.7
277		9.579	0.1	48.7	0.3	50.9

278		9.939	0.3	20.7	0.4	78.7
279		9.983	17.8	2.3	79.6	0.3
280		10.11	15.7	40.2	28.7	15.5
281		10.194	13.9	2.3	83.4	0.4
282		10.267	9.1	26.4	28.1	36.4
283		10.335	4.8	12.6	38.4	44.2
284		10.39	3	15	7.8	74.2
285		10.438	14.4	42.9	22.5	20.1
286		10.453	19.1	19.4	24.2	37.3
287		10.519	20	6.4	31.7	41.9
288		10.557	14.7	11.6	27.5	46.2
289		10.626	7.9	0.8	90.1	1.1
290		10.693	18.7	13	42.2	26
291		10.785	2.4	32.2	4.6	60.9
292		10.949	32.7	9.2	37.6	20.6
293		11.031	15.1	17	38.2	29.7
294		11.135	26	11.7	46.6	15.8
295		11.268	1.8	49	2.2	47
296		11.284	12.1	34.3	14.4	39.2
297		11.351	32.9	11.3	43.5	12.2
298		11.571	0.2	40.9	0.5	58.3
299		11.704	42.3	0.1	57.5	0.1
300		11.814	28.6	0.2	71.1	0.1
301		11.949	0	38.8	0	61.1
302		12.066	40.9	0.1	59	0.1
303		12.229	1.7	54	1.7	42.7
304		12.364	59.6	2.8	35.3	2.2
305		12.509	7.1	39.7	7.3	46
306		12.567	1.4	53.3	1.3	44.1
307		12.594	14.3	34.6	13.1	38
308		12.629	29.8	18.7	30.8	20.8
309		12.649	51.9	1.3	45.3	1.6
310		12.822	48.6	7.8	37.5	6.2
311		12.854	8.2	48.5	6.7	36.6
312		12.955	0	42.1	0	57.8
313		12.982	43.8	0	56.1	0
314		13.148	36.9	0	63	0.1
315		13.215	0.2	36.6	0.5	62.6
316		13.275	39.3	0.3	60.1	0.3
317		13.535	0	32.1	0	67.9
318		13.764	29.6	0.1	70.3	0.1
319		13.899	0.4	38.5	0.7	60.5
320		13.912	0.4	35.5	0.8	63.3

321		14.068	26.7	3.2	64.2	5.9
322		14.229	5	17.6	10.8	66.7
323		14.258	13.4	11.1	48	27.5
324		14.27	15	6.7	64.7	13.6
325		14.376	0.4	32.3	1.1	66.2
326		14.439	26.9	0.4	71.8	0.8
327		14.698	19.3	19.8	31.6	29.2
328		15.174	0	23.7	0	76.3
329		15.513	2.3	18.4	6.5	72.7
330		15.532	23.5	1.8	67.7	7
331		15.736	19.4	0	80.4	0.1
332		15.879	30.6	0	69.3	0.1
333		15.89	0	29.5	0	70.4
334		16.1	9.6	21.5	23.2	45.7
335		16.117	20.5	10.7	46.9	21.9
336		17.098	36	0.4	62.8	0.7
337		17.174	15.4	20.6	27.1	37
338		17.259	21.4	14.6	37.6	26.3
339		17.437	0	29.1	0	70.9
340		17.48	0	20	0	79.9
341		17.564	9.2	0	90.8	0
342		18.13	0	9	0	91
343		18.241	8.6	0	91.4	0
344		18.288	0	22.2	0.1	77.7
345		18.369	21.2	0	78.8	0
346		20.32	0	4.4	0	95.6
347		20.396	4.3	0	95.7	0
348		20.571	0	6.1	0	93.9
349		20.677	6	0	94	0

Table S11: Electronic transition of Cd-NDI complex through CAM-CAM-B3LYP/LANL2DZ.

**Transition**	**Energy J/mol**	**Translation**	**Energy J/mol**
$\sigma_{O4-C21} \rightarrow n^{*}(6)_{Cd1}$	0.57	$\pi_{O53-C75} \rightarrow \pi^{*}_{C71-C89}$	4.83
$\sigma_{O4-C21} \rightarrow n^{*}(7)_{Cd1}$	1.08	$\sigma_{C43-H44} \rightarrow \sigma^{*}_{C13-C25}$	2.06
$\sigma_{C15-H16} \rightarrow \sigma^{*}_{C27-C32}$	7.7	$\sigma_{C15-H16} \rightarrow \sigma^{*}_{C12-C13}$	6.62
$\pi_{C25-C43} \rightarrow \pi^{*}_{C20-C30}$	9.03	$\sigma_{C30-H31} \rightarrow \sigma^{*}_{C20-C22}$	6.59
$\pi_{C12-C15} \rightarrow \pi^{*}_{C27-C32}$	8.98	$\sigma_{C15-H16} \rightarrow \sigma^{*}_{C20-C22}$	6.59
$\pi_{C27-C32} \rightarrow \pi^{*}_{O6-C26}$	7.5	$\sigma_{C27-H28} \rightarrow \sigma^{*}_{C22-C32}$	6.53
$\pi_{C25-C43} \rightarrow \pi^{*}_{O7-C29}$	5.07	$\sigma_{C35-H37} \rightarrow \sigma^{*}_{N11-C33}$	6.07
$\pi_{O52-C72} \rightarrow \pi^{*}_{C73-C78}$	4.98	$\sigma_{C39-H41} \rightarrow \sigma^{*}_{N8-C17}$	6.07
$\pi_{O9-C19} \rightarrow \pi^{*}_{C20-C30}$	4.97	$\sigma_{C33-H34} \rightarrow \sigma^{*}_{N11-C26}$	5.79
$\pi_{O7-C29} \rightarrow \pi^{*}_{C25-C43}$	4.95	$\sigma_{C17-H18} \rightarrow \sigma^{*}_{N8-C29}$	5.57
$\pi_{O51-C69} \rightarrow \pi^{*}_{C58-C61}$	4.94		
$\pi_{O5-C23} \rightarrow \pi^{*}_{C12-C15}$	4.86		

Table S12: Electronic transition of Cd-NDI complex through B3LYP/LANL2MB.

Transitions	Energy (J/mol)	Transitions	Energy (J/mol)
σ $_{N11-C33}$→n*(9) $_{Cd1}$	0.07	σ $_{O6-C26}$→σ* $_{N11-C26}$	1.91
σ $_{O14-C21}$→n*(6) $_{Cd1}$	1.05	σ $_{O6-C26}$→σ* $_{C22-C32}$	0.66
σ $_{C35-H37}$→n*(7) $_{Cd1}$	0.13	σ $_{O6-C26}$→σ* $_{C26-C32}$	1.2
σ $_{C35-H38}$→n*(7) $_{Cd1}$	0.15	π $_{O6-C26}$→π* $_{O6-C26}$	2.98
σ $_{O2-H3}$→σ* $_{O2-C24}$	0.77	π $_{O6-C26}$→π* $_{C27-C32}$	4.79
σ $_{O2-H3}$→σ* $_{O10-C24}$	3.79	σ $_{O7-C29}$→σ* $_{N8-C23}$	0.73
σ $_{O2-H3}$→σ* $_{C17-C24}$	2.48	σ $_{O7-C29}$→σ* $_{N8-C29}$	1.95
σ $_{O2-C24}$→σ* $_{N8-C17}$	0.66	σ $_{O7-C29}$→σ* $_{C13-C25}$	0.66
σ $_{O2-C24}$→σ* $_{O10-C24}$	2.71	σ $_{O7-C29}$→σ* $_{C25-C29}$	1.24
σ $_{O2-C24}$→σ* $_{C17-C24}$	2.99	π $_{O7-C29}$→π* $_{O7-C29}$	0.69
σ $_{O2-C24}$→σ* $_{C17-C39}$	0.52	π $_{O7-C29}$→π* $_{C25-C43}$	5.91
σ $_{O4-C21}$→σ* $_{O14-C21}$	2.13	σ $_{N8-C17}$→σ* $_{O2-H3}$	0.73
σ $_{O4-C21}$→σ* $_{C21-C33}$	1.9	σ $_{N8-C17}$→σ* $_{O2-C24}$	0.73
σ $_{O5-C23}$→σ* $_{N8-C23}$	1.93	σ $_{N8-C17}$→σ* $_{O5-C23}$	1.62
σ $_{O5-C23}$→σ* $_{N8-C29}$	0.73	σ $_{N8-C17}$→σ* $_{O7-C29}$	1.56
σ $_{O5-C23}$→σ* $_{C12-C13}$	0.65	σ $_{N8-C17}$→σ* $_{N8-C23}$	2.27
σ $_{O5-C23}$→σ* $_{C12-C23}$	1.25		
σ $_{O6-C26}$→σ* $_{N11-C19}$	0.76		

Table S13: Comparison on NLO properties of present work with already reported work.

Complex	Method	αiso (A.U.)/ x10^{-22}esu	Averages second harmonic generation β(-2w;w,w)a(a.u.)/ x10^{-30} esu	Second order hyperpolarizability or SHG or γ (A.U.)/ esu x10^{-36}	References
Cd-NDI	CAM-B3LYP/LANL2DZ	705/1.04	7967/68.8	1000000000/5036.7	Present work
Cd-NDI	B3LYP/LANL2MB	465/0.7	78964/682.1	363564000/1831.1	Present work
H_2-L-ala NDI	CAM-B3LYP/LANL2DZ	504/0.7	2000/17.2	16993000/85.6	Present work
KDP	CAM-B3LYP/LANL2DZ	41.8249/0.06	2766/23.9	1363640/6.9	Present work
Urea	CAM-B3LYP/LANL2DZ	26/0.004	10/0.009	1858/0.01	Present work
NDI derivative	CAM-B3LYP	1141.0/1.7	NA	188215.6/ 0.9	[1]
NDI-COR	CAM-B3LYP	913.4/1.4	NA	274907.7/1.4	[1]
NDI-2COR	CAM-B3LYP	728.6/1.1	NA	392069.9/2.0	[1]
[Os(CO)$_3$Cl2L1]	experimental	NA	43 esu	NA	[2]
ZnTFP-L2	experimental	NA	2.42 esu	NA	[3]
ZnTPP-L2	experimental	NA	0.74 esu	NA	[3]
ZnTBP-L2	experimental	NA	2.34 esu	NA	[3]
ZnTNP-L2	experimental	NA	1.71 esu	NA	[3]
Cd^{2+}-Complex	experimental	NA	NA	4.1 γ(X10-44m^5/V^2)	[4]
NDI-2NO_2	CAM-B3LYP	NA	NA	62.43000 esu	[5]
Ni-Schiff-base	experimental	NA	235 esu	NA	[6]
Cu-Schiff-base	experimental	NA	237 esu	NA	[6]

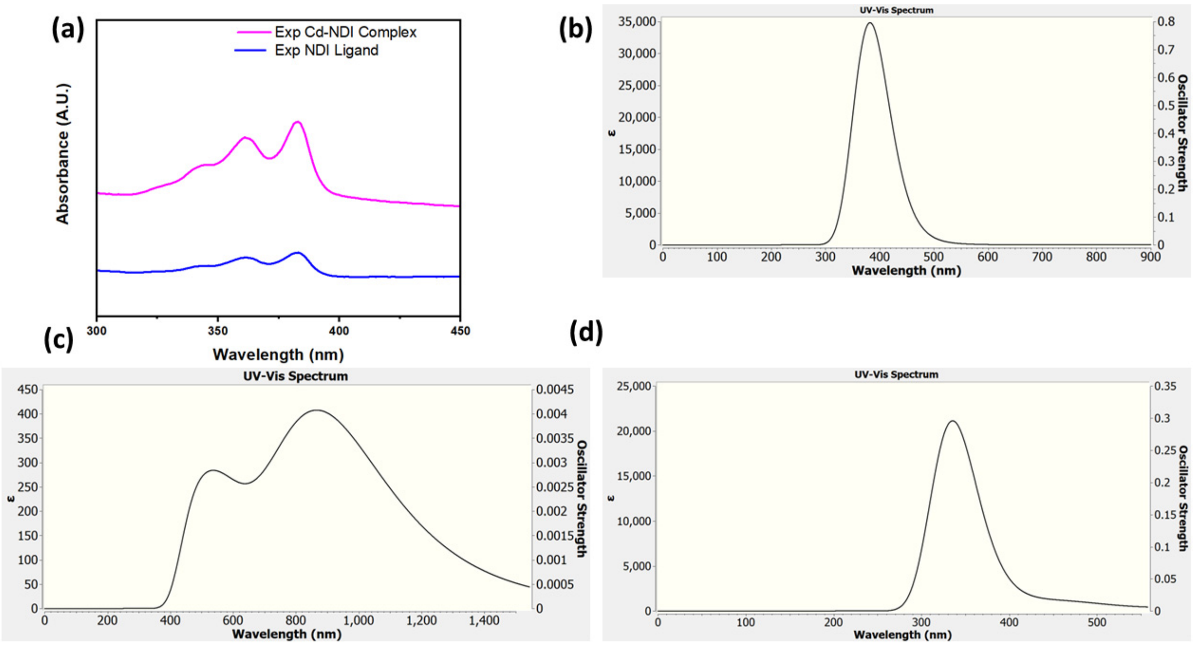

Figure S4: UV-visible absorption graphs of (a) experimental Cd-NDI complex, (b) computational of Cd-NDI complex through CAM-B3LYP/LANL2DZ (c) computational of Cd-NDI complex through B3LYP/LANL2MB and (d) computational of H_2-L-ala NDI through CAM-CAM-B3LYP/LANL2DZ, respectively.

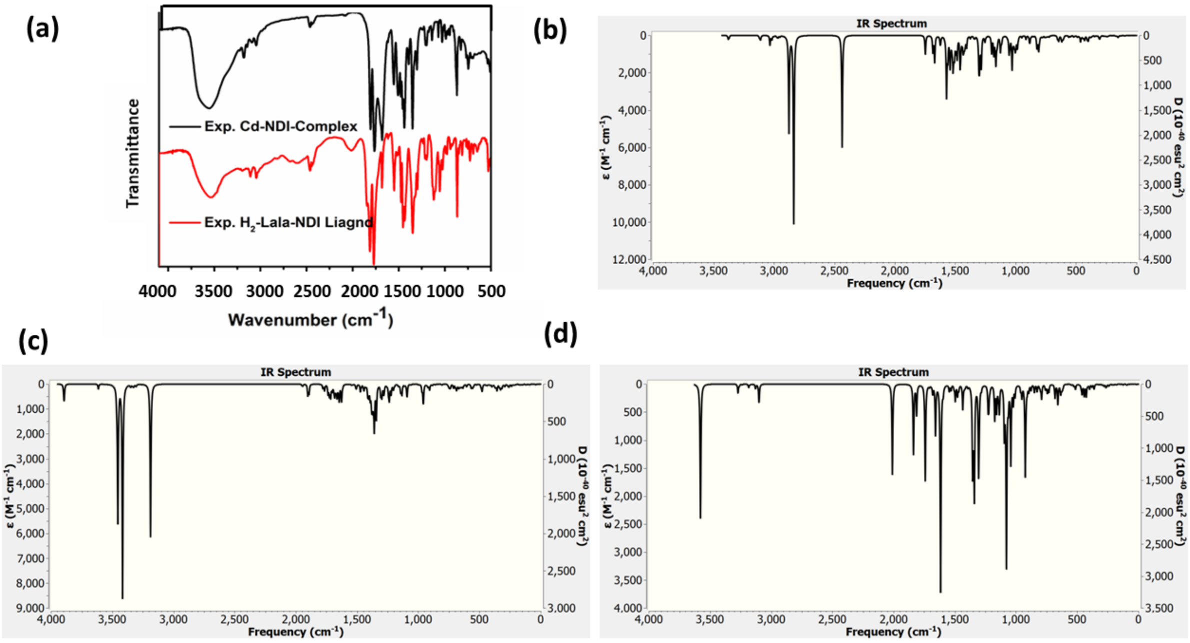

Figure S5: IR spectra of (a) experimental Cd-NDI complex, (b) computational of Cd-NDI complex through CAM-CAM-B3LYP/LANL2DZ (c) computational of Cd-NDI complex through B3LYP/LANL2MB and (d) computational of H_2-L-ala NDI through CAM-CAM-B3LYP/LANL2DZ, respectively.

References

1. Dolomanov, O.V.; Bourhis, L.J.; Gildea, R.J.; Howard, J.A.; Puschmann, H. OLEX2: A complete structure solution, refinement and analysis program. J. Appl. Crystallogr. 2009, 42, 339–341
2. Wang, L.; Liu, Y.-L.; Wang, M.-S. The organic co-crystals formed using naphthalenediimide-based triangular macrocycles and coronene: intermolecular charge transfers and nonlinear optical properties. *Phys. Chem. Chem. Phys.* **2022**, *24*, 29747–29756, https://doi.org/10.1039/d2cp03236h.
3. Lucenti, E.; Cariati, E.; Dragonetti, C.; Manassero, L.; Tessore, F. Effect of the Coordination to the "Os3 (CO) 11" Cluster Core on the Quadratic Hyperpolarizability of trans-4-(4 ′-X-styryl) pyridines (X= NMe2, t-Bu, CF3) and trans, trans-4-(4 ′-NMe2-phenyl-1, 3-butadienyl) pyridine.*Organometallics* **2004**, *23*, 687–692. https://doi.org/10.1021/om034086r
4. Tessore, F.; Di Carlo, G.; Forni, A.; Righetto, S.; Limosani, F.; Biroli, A.O. Second Order Nonlinear Optical Properties of 4-Styrylpyridines Axially Coordinated to A_4 Zn^{II} Porphyrins: A Comparative Experimental and Theoretical Investigation. *Inorganics* **2020**, *8*, 45, https://doi.org/10.3390/inorganics8080045.
5. Prasad, G.K.; Prashanth, S.; Srivastava, S.; Rao, G.N.; Babu, D.R. Synthesis, characterization, second and third order non-linear optical properties and luminescence properties of 1,10-phenanthroline-2,9-di(carboxaldehyde phenylhydrazone) and its transition metal complexes. *Open Chem.* **2017**, *15*, 283–292, https://doi.org/10.1515/chem-2017-0036.
6. Qiu, Y.-Q.; Li, Z.; Ma, N.-N.; Sun, S.-L.; Zhang, M.-Y.; Liu, P.-J. Third-order nonlinear optical properties of molecules containing aromatic diimides: Effects of the aromatic core size and a redox-switchable modification. *J. Mol. Graph. Model.* **2013**, *41*, 79–88.
7. Trujillo, A.; Fuentealba, M.; Carrillo, D.; Manzur, C.; Ledoux-Rak, I.; Hamon, J.-R.; Saillard, J.-Y. Synthesis, spectral, structural, second-order nonlinear optical properties and theoretical studies on new organometallic donor− acceptor substituted nickel (II) and Copper (II) unsymmetrical Schiff-base complexes. *Inorg. Chem.* **2010**, *49*, 2750–2764.
